# Importin β Can Bind Hepatitis B Virus Core Protein and Empty Core-Like Particles and Induce Structural Changes

**DOI:** 10.1371/journal.ppat.1005802

**Published:** 2016-08-12

**Authors:** Chao Chen, Joseph Che-Yen Wang, Elizabeth E. Pierson, David Z. Keifer, Mildred Delaleau, Lara Gallucci, Christian Cazenave, Michael Kann, Martin F. Jarrold, Adam Zlotnick

**Affiliations:** 1 Department of Molecular and Cellular Biochemistry, Indiana University, Bloomington, Indiana, United States of America; 2 Department of Chemistry, Indiana University, Bloomington, Indiana, United States of America; 3 Universite de Bordeaux, Microbiologie Fondamentale et Pathogénicité, UMR 5234, Bordeaux, France; 4 CNRS, Microbiologie Fondamentale et Pathogénicité, UMR 5234, Bordeaux, France; 5 CHU de Bordeaux, Bordeaux, France; 6 Department of Biology, Indiana University, Bloomington, Indiana, United States of America; The Pennsylvania State University College of Medicine, UNITED STATES

## Abstract

Hepatitis B virus (HBV) capsids are found in many forms: immature single-stranded RNA-filled cores, single-stranded DNA-filled replication intermediates, mature cores with relaxed circular double-stranded DNA, and empty capsids. A capsid, the protein shell of the core, is a complex of 240 copies of core protein. Mature cores are transported to the nucleus by a complex that includes both importin α and importin β (Impα and Impβ), which bind to the core protein’s C-terminal domains (CTDs). Here we have investigated the interactions of HBV core protein with importins in vitro. Strikingly, empty capsids and free core protein can bind Impβ without Impα. Cryo-EM image reconstructions show that the CTDs, which are located inside the capsid, can extrude through the capsid to be bound by Impβ. Impβ density localized on the capsid exterior near the quasi-sixfold vertices, suggested a maximum of 30 Impβ per capsid. However, examination of complexes using single molecule charge-detection mass spectrometry indicate that some complexes include over 90 Impβ molecules. Cryo-EM of capsids incubated with excess Impβ shows a population of damaged particles and a population of “dark” particles with internal density, suggesting that Impβ is effectively swallowed by the capsids, which implies that the capsids transiently open and close and can be destabilized by Impβ. Though the in vitro complexes with great excess of Impβ are not biological, these results have implications for trafficking of empty capsids and free core protein; activities that affect the basis of chronic HBV infection.

## Author Summary

Viruses take advantage of host proteins. During infection, DNA-filled Hepatitis B Virus (HBV) cores are ferried to the nucleus by a complex of importin α and importin β; importinβ alone does not bind mature cores. However, a surprisingly large amount of the HBV core protein accumulates in nuclei. Here we show that both empty cores and free core protein can bind importin β alone. Using cryo-electron microscopy reconstructions we show that importin β associates with a putative importin β-binding motif, which is located on the interior of empty cores but can extrude through pores in the particle. Single-particle mass spectrometry, electron microscopy, and biochemical studies showed the importin β can destabilize empty particles. These results together demonstrate unsuspected interactions between the HBV core protein and the nuclear transport system. These interactions may have roles in regulating stability of empty capsids and transfer of HBV core protein to the nucleus.

## Introduction

Viruses take advantage of host proteins throughout their lifecycle; here we investigate interactions between Hepatitis B Virus (HBV) and host proteins related to nuclear transport. HBV is an enveloped virus with an icosahedral core. Mature HBV cores, which consist of a relaxed circular dsDNA genome packaged in a protein capsid, arrive in the cytoplasm as newly infecting particles or by maturation of newly assembled RNA-filled cores. In chronically infected cells, cores form in the cytoplasm when the HBV core (or capsid) proteins assemble around a complex of HBV reverse transcriptase and an RNA transcript of the viral genome. A large fraction of core protein assembles into empty capsids, devoid of RNA [1]. The fraction of empty capsids appears to increase in response to treatment of infected cells with TNFα or small molecules (i.e. core protein allosteric modulators) that drive capsid assembly [2,3]. Mature cores are transported to nuclear pores by a complex of importin α and importin β [4,5]. In a chronically infected cell, mature cores and empty cores, but not immature ssDNA-filled cores, can also be secreted from the host cell, acquiring an HBV surface antigen-studded envelope in the process [6,7].

**Fig 1:**
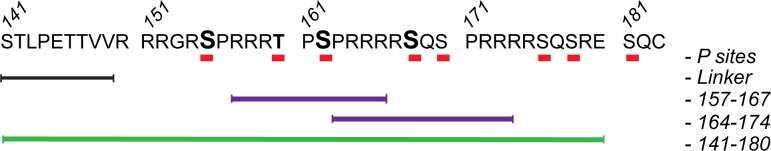
The C-terminal domain (CTD) of HBV core protein. Cp183 is comprised of an assembly domain (residues 1–149) that includes a nine-residue linker (underlined by the black line, 141–149) [83], and a 34-residue CTD. The CTD has seven phosphorylatable serines and one phosphorylatable threonine (red lines). Phosphorylation of serines 155, 162, and 170 (large letters) appears to play a critical role in RNA-packaging [11]. Arginine-rich segments have been associated with nuclear localization signals [21–23,27]; as an example, two peptides that conform to a classical bipartite NLS sequences (purple lines) have been demonstrated to compete with known NLS sequences [8]. Cp183’s IBB segment has not been specifically identified but an IBB is ~40 amino acids including ~17 basic residues [28] and could thus incorporate most of the CTD as well as the linker region (green line); no other sequence in Cp183 has this wealth of basic residues.

The predominant form of capsid is a complex of 120 core protein dimers arranged with T = 4 icosahedral symmetry. The core protein, herein referred to as Cp183, has 183 amino acids (185 for certain genotypes), forming two distinct domains: a helix-rich 149-residue assembly domain, capable of forming capsids on its own, and a disordered 34-residue, basic, nucleic acid-binding, C terminal domain (CTD) (Fig 1). The CTD can be phosphorylated at seven serines and one threonine [8–10]. The CTD phosphorylation is necessary for correct RNA packaging, affects reverse transcription, and can modulate nuclear transport [4,10–12]. In capsids, the CTD is localized to the interior interacting with packaged RNA or DNA [13], but genome maturation and CTD phosphorylation lead to at least transient external exposure [4,8,14]. Indeed, cytoplasmic cores can show evidence of proteolysis of the encapsidated reverse transcriptase, implying at least transient opening of the capsids [15]. In empty capsids, CTDs readily extrude from capsid holes where they can be bound by external molecules [16] or cleaved by a protease [17].

Transport of HBV cores across the nuclear envelope is via nuclear pore complexes [18]. Genome liberation is closely linked with core interaction with nuclear pores [19,20]–crosslinked capsids become trapped in the nuclear pore [14]. Nuclear transport of HBV is mediated by cellular transport receptors, termed importins or karyopherins [8,9,21–23]. Canonically, the adapter protein importin α (Impα) binds to a nuclear localization sequence (NLS), exposing Impα’s importin β-binding sequence (IBB) which in turn binds to the transport protein importin β (Impβ) [24]. An NLS consists of one or two clusters of four to six basic amino acids [25]. Interactions between importins and with cargo depend on electrostatic forces. HBV cores bind importins via CTD-associated NLS sequences [8,9,21–23,26,27]. Impα is required for transport of mature HBV cores to the nucleus [4,14]. As an alternative to Impα-mediated transport, Impβ can bind directly to cargos that have an IBB; a canonical IBB is comprised of 13 basic amino acids in 7 clusters distributed over 39 residues [28,29]. The Cp183 CTD may also contribute to forming an IBB (Fig 1).

The intracellular fate of an HBV core or capsid, e.g. its nuclear transport, appears to be a function of its nucleic acid content. In this regard, there are four different forms of capsids in infected cells: RNA-filled immature, replication intermediate-containing immature, rcDNA-filled mature, and empty. Empty capsids show structural similarities to mature capsids [30] and like mature can become enveloped by the surface proteins [1]. The predominant localization of capsids varies: HBV-expressing transformed cell lines [31] and infected primary hepatocytes in culture show a cytosolic dominant phenotype, while infected hepatocytes from patients’ livers or in HBV transgenic mice mostly exhibit nuclear capsids [32–34]. Of note, cytosolic cores in patients are linked to greater hepatocellular injury [35,36]. Cytoplasmic expression of hepatitis B core antigen correlates with histologic activity of liver disease in young patients with chronic hepatitis B infection [36,37]. This is consistent with the observation that core epitopes, derived from proteasomal degradation of the core or of core protein dimers [38] are a target for the MHC class I mediated CD8+ T cell response, which can in turn modulate HBV infection [39]

We have used a reductionist system comprised of Impβ and Cp183 to investigate the basis of importin-core protein interaction. We find that Impβ can bind phosphorylated and unphosphorylated Cp183, Impβ binds Cp183 dimers and Cp183 empty capsids, and excess Impβ can spontaneously destabilize capsids. These results suggest that the combination of a dynamic capsid and a ligand like Impβ, whose binding site extends into the assembly domain, can provide a mechanical basis for initiating HBV uncoating. This activity may have roles in genome release and clearing empty capsids from the cytoplasm.

## Results

### Importin β (Impβ) binds to HBV capsids

**Fig 2:**
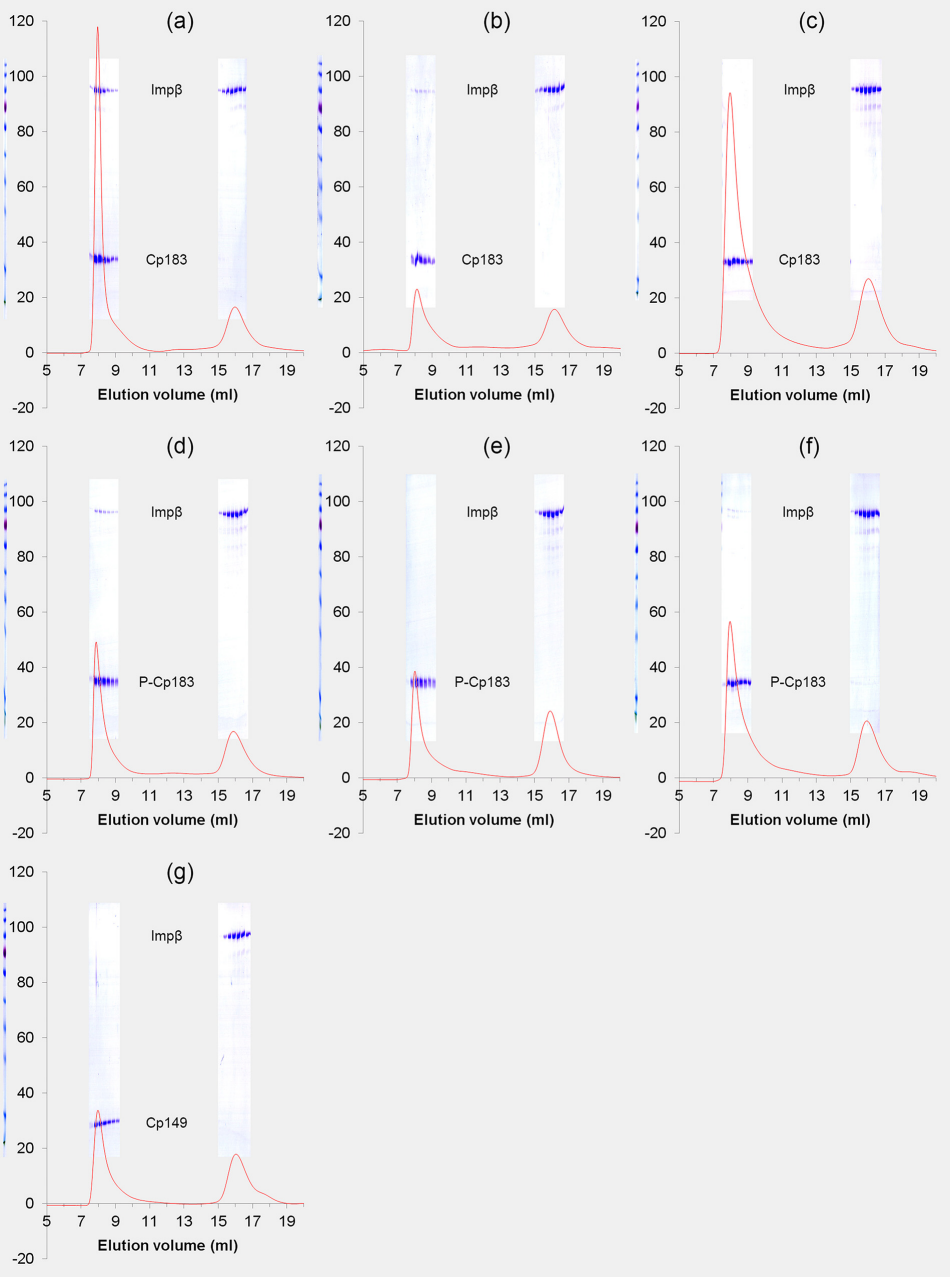
Impβ binding to HBV is affected by ionic strength and requires the arginine rich C-terminus. Elution profiles of human importin **β**1 (Impβ, 5.3 μM) with HBV capsids (7.9 μM dimer). Samples of Impβ with (a) empty Cp183 capsids, 0.15 M NaCl; (b) empty Cp183 capsids, 0.25 M NaCl; (c) Cp183 capsids containing *E*. *Coli* RNA, 0.15 M NaCl: (d) empty P-Cp183 capsids, 0.15 M NaCl; (e) empty P-Cp183 capsids, 0.25 M NaCl; (f) P-Cp183 capsids containing *E*. *Coli* RNA, 0.15 M NaCl: (g) empty Cp149 capsids, 0.15 M NaCl. Samples were eluted through a Superose 6 column equilibrated with 20 mM TrisHCl pH7.4 and NaCl at the came concentration used for sample incubation and at a flow rate of 0.5 ml/min. Chromatographs are overlaid by images of Coomassie-stained SDS-PAGE of the corresponding fractions.

HBV CTDs are normally on the capsid interior where they bind nucleic acid [40,41], but can be transiently exposed and captured on the capsid exterior by a binding partner [8,14,16,17]. We initially examined Cp183 capsids binding to Impα and Impβ, separately and together, using size exclusion chromatography (SEC, Fig 2). Though mature capsids had been found to require the Impα adapter protein to bind Impβ [8], we observed that Cp183 capsids co-migrated with Impβ and both Impα and Impβ. We chose to focus on the interaction of Cp183 with Impβ (without Impα) for three reasons. First, we observed that the binding affinity of Impβ for empty capsids was about the same as that of the Impα+Impβ complex, which suggest direct impβ binding may be biologically relevant for empty capsids. Second, the difference in binding specificity of mature and immature cores suggests the hypothesis that there could be discrete structural or dynamic differences between them. Third, a practical issue arose: we needed to fully understand Cp183-Impβ interaction before investigating binding to an Impα+Impβ complex. Finally, up until very recently the prevalence of empty capsids in HBV infections had not been appreciated [1].

To determine the requirements for Impβ interaction with cores, we tested different forms of capsid by monitoring the co-migration of Impβ and capsids through a Superose 6 column (Fig 2, S1 Table). In these assays the concentrations of the reactants, reaction conditions, and column conditions were kept identical to allow valid comparison of the Impβ affinities. The reactant concentrations were 5.3 μM Impβ and 7.9 μM core protein dimer (equivalent to 66 nM capsid); this resulted in a molar ratio of 80 Impβ per capsid, an excess over the 30 sites anticipated from earlier studies with CTD-binding SRPK [16].

The elution profiles (e.g., Fig 2A) show that Impβ binds to empty Cp183 capsids in solution. Increasing the NaCl concentration from 0.15 M to 0.25 M notably decreased binding Impβ to Cp183 capsid demonstrating the electrostatic nature of the interaction between Cp183 capsids and Impβ (Fig 2B vs. Fig 2A). We observed that Cp183 capsids that contained *E*. *coli* RNA, predicted to entrap CTDs on the capsid interior, did not detectably bind Impβ using Coomassie staining (Fig 2C), though a small population of bound Impβ could be detected by silver staining. Capsids of Cp149, a core protein lacking the basic CTD, did not measurably bind Impβ (Fig 2G). These data are consistent with localization of the Cp183 NLS/IBB segments to the proteins’ C-terminal domains (CTDs) [21,23,27].

Phosphorylation of NLS sequences can up- and down-regulate nuclear transport and alter importin binding [42]. We investigated how the phosphorylation affected HBV capsids’ affinity for Impβ by performing the column assays on phosphorylated Cp183 (P-Cp183) capsids (Fig 2D and 2E). P-Cp183 was prepared by co-expressing Cp183 and the protein kinase SRPK1 in *E*. *coli* [43]. The phosphorylation status of the capsids with or without RNA was characterized by mass spectrometry (S2 Fig). The affinity of Impβ was much weaker for P-Cp183 than for unphosphorylated Cp183 (compare Fig 2D with 2A). In the presence of 0.25 M NaCl, Impβ binding was further suppressed (Fig 2E); a small population of Impβ bound to P-Cp183 capsids could be observed in silver stained SDS-PAGE.

Despite the lower affinity of empty P-Cp183 capsids for Impβ compared to empty Cp183 capsids, P-Cp183 capsids incorporating *E*. *coli* RNA bound more Impβ than RNA-filled Cp183 capsids (Fig 2F vs. Fig 2C). This result is consistent with earlier observations suggesting that phosphorylated CTDs would be less restrained by negatively-charged RNA than un-phosphorylated CTDs [8].

In summary, we observed: (i) Empty HBV capsids can bind to Impβ directly without Impα. (ii) The binding sites are associated with CTDs and the binding interaction has an electrostatic nature. (iii) Phosphorylation of the CTD reduces its interaction with Impβ and, presumably, encapsidated nucleic acid.

### Cryo-EM image reconstruction of intact capsids decorated with Impβ

**Fig 3:**
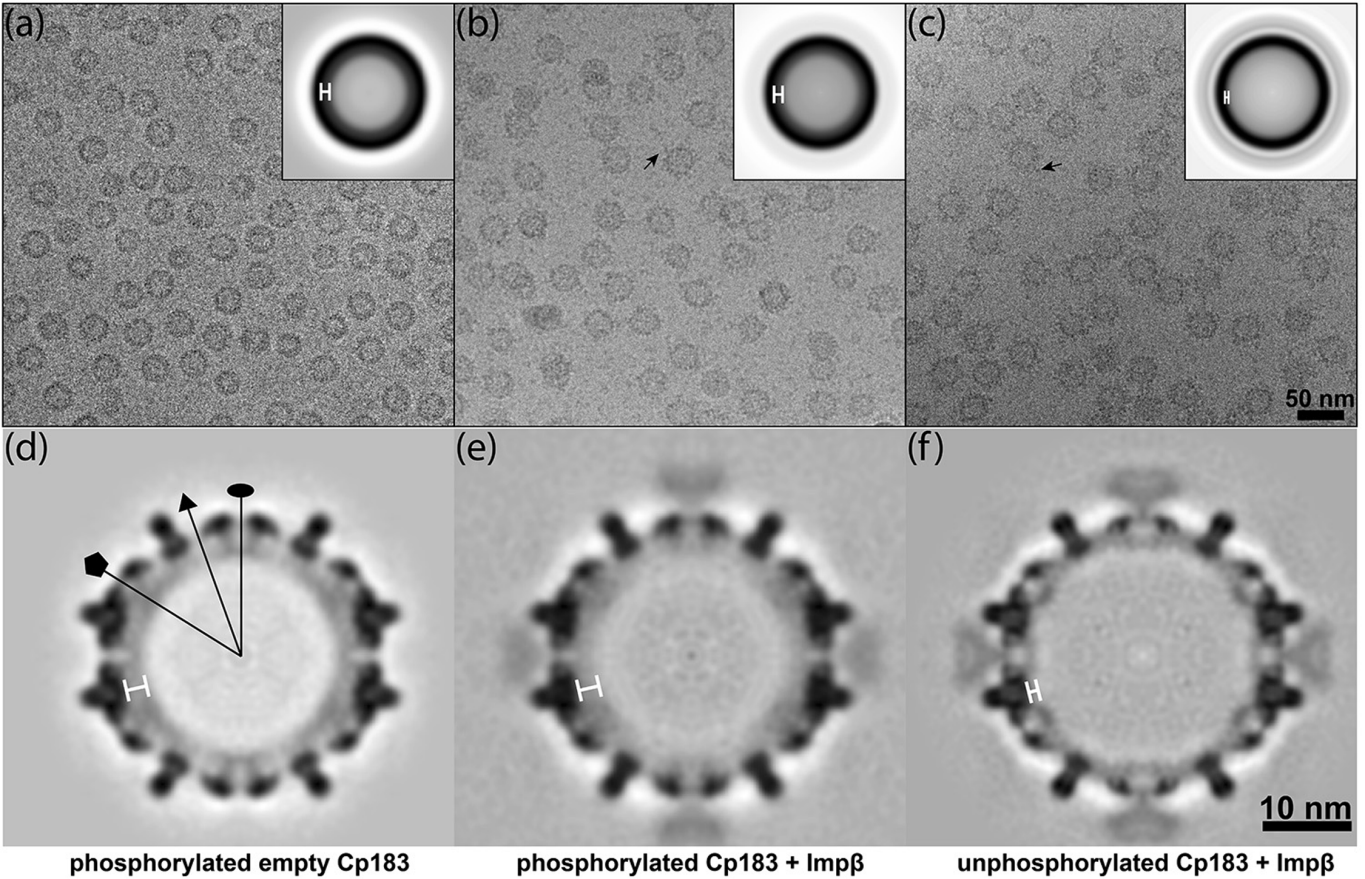
Cryo-EM analysis of Cp183 capsids complexed with Impβ. Typical cryo-EM micrographs of (a) P-Cp183, (b) P-Cp183-Impβ, and (c) Cp183-Impβ and (d-f) central sections of their respective reconstructions. Insets in the micrographs show enlarged, translationally and rotationally aligned average images. Black arrows in the micrographs point to examples of Impβ decorating the capsids. In reconstructions, the Impβ is found at the twofold vertices on the Cp183 capsid surface. CTDs are localized to the annulus indicated by the white bars in the rotationally aligned images and the density maps. The P-Cp183 capsids show stronger CTD density at their fivefold vertices consistent with clustering of CTDs previously described in reference [13]. The capsid-Impβ complexes were prepared from Cp183 capsid (7.9 μM dimer) and 5.3 μM Impβ. Oval, triangle, and pentagon symbols in panel d indicate locations of twofold, threefold and fivefold axes, respectively. The resolution of the maps shown in panels d-f are 10.1 Å, 10.9 Å, and 10.2 Å, respectively, based on Fourier Shell Correlation (FSC) gold standard methodology using an FSC cutoff of 0.143 (S2 Table).

As discussed below, cryo-electron microscopy (cryo-EM) structural studies revealed localization of Impβ and showed that the importin and CTDs were highly disordered. Impβ-decorated capsids (P-Cp183-Impβ and Cp183-Impβ) were prepared for cryo-EM from a mixture of 5.3 μM Impβ and 7.9 μM Cp183 dimer, corresponding to a ratio of 80 Impβ proteins per capsid. In the absence of Impβ, control micrographs showed that empty P-Cp183 capsids and empty unphosphorylated Cp183 capsids were very similar (compare Fig 3A in this paper to Figure 2a and 2b in reference [13]). Cryo-EM micrographs of Impβ-decorated capsids showed capsids festooned by sporadic protrusions of additional density (Fig 3B and 3C, black arrows). In translationally aligned images of Impβ-decorated capsids we observed an additional ring located outside the capsid layer. This outer ring of density is notably stronger for unphosphorylated Cp183-Impβ capsids than phosphorylated particles (Fig 3B and 3C, insets).

We computed icosahedrally averaged 3D reconstructions from 16,591 particle images for empty P-Cp183, 5,715 particle images for P-Cp183-Impβ, and 8,513 particles for Cp183-Impβ (Fig 3). Final resolutions were estimated to be 10.1 Å, 10.9 Å, and 10.2 Å, respectively, based on a Fourier shell correlation threshold of 0.5 (S2 Table). All three structures exhibited clearly defined features typical for T = 4 HBV (Fig 3D, 3E and 3F and S3 Fig). Dimer spikes were sufficiently resolved to show the component α helices. An atomic HBV capsid structure (PDB entry 1QGT [44]) fits the cryo-EM density attributable to capsid with no modification (S3 Fig). In addition to the density expected for a capsid, flower-shaped density was observed protruding from the holes at quasi-sixfold vertices in both P-Cp183-Impβ and Cp183-Impβ structures (Fig 3E and 3F), where the capsid protein CTD can extrude. This density was much weaker than protein density, suggesting only partial occupancy and/or heterogeneous binding modes. We attribute this new density to bound Impβ, agreeing the conclusion that Impβ binds to CTD. Further, we calculated a difference map by subtracting an empty P-Cp183 capsid structure from a P-Cp183-Impβ structure, generating surface shaded densities matching the molecular model of Impβ (PDB entry 3LWW [45]) (S3 Fig). It is clear from the density and model that there is close contact between a single Impβ and the surrounding spikes at a quasi-sixfold vertex, despite the availability of six CTDs around the location. Therefore, we conclude that there is room for only one Impβ at each quasi-sixfold location, leading to a maximum of 30 sites per capsid.

### Charge-Detection Mass Spectrometry revealed complexes of Cp183 associated with more than 50 Impβ molecules

**Fig 4:**
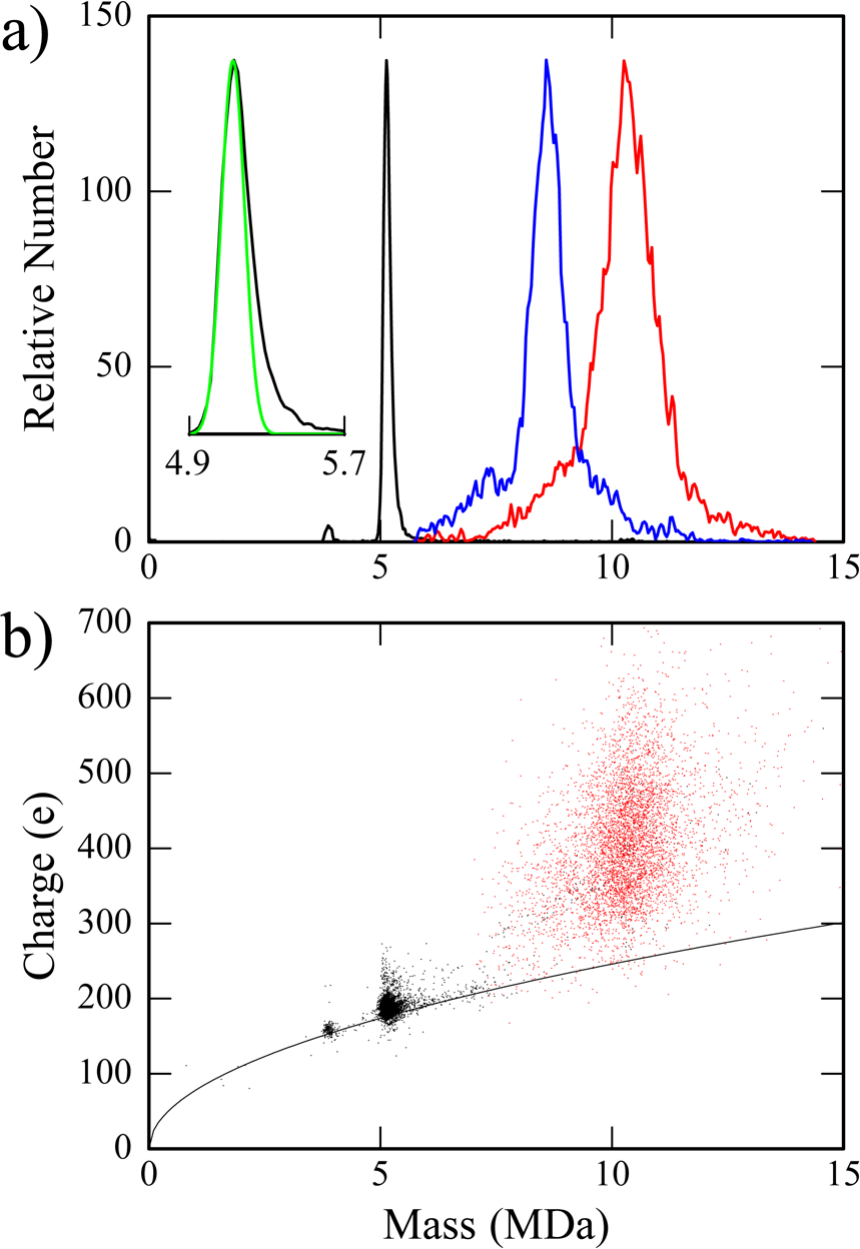
CDMS of Impβ complexes with Cp183 capsids show heterogeneity and flexibility. (a) CDMS mass spectra of Cp183 capsids (7.9 μM dimer, 66 nM capsid) and capsids with Impβ. Without Impβ we observe a single peak (inset), centered at the expected mass of 5.1 MDa but with a broad high mass tail (black); the green line shows the expected Gaussian peak shape. Spectra for Cp183 capsid (7.9 μM dimer, 66 nM capsid) with 3.0 μM Impβ (blue) and 14.8 μM Impβ (red), 45:1 and 224:1 Impβ:capsid, respectively, show heterogeneous capsid-Impβ complexes. At the highest concentration of Impβ, masses extend to about 15 MDa, consistent with capsid decorated with more than 90 Impβ molecules. The CDMS spectrum for the bare Cp183 capsid shown in panel a was generated from 8943 ions were binned in 20 kDa bins. For Cp183 with 3.0 and 14.8 μM Impβ, 5644 and 5772 ions, respectively, were binned in 40 kDa bins. Histograms were smoothed with a five point Savitsky-Golay algorithm. (b) A plot of charge versus mass for the data in panel (a). The black line shows the predictions of the charge residue model discussed in the text. Cp183 capsids (black points) have a narrow distribution consistent with an object with a well-defined geometry. Cp183 capsids with 14.8 μM Impβ (red) show a much more disperse distribution of charge. The bare capsid sample in the inset was dissolved in 250mM ammonium formate; samples with Impβ were in 150mM ammonium formate.

To definitively determine the distribution of Impβ bound to HBV capsids, we used charge detection mass spectrometry (CDMS), a single particle MS technique. Unlike conventional MS techniques that measure the mass to charge ratio (m/z) for an ensemble of ions and require charge state deconvolution to determine z and then m, CDMS simultaneously measures m/z and z for each ion, yielding m directly. This enables the analysis of very large and heterogeneous species that resist conventional MS analysis [46]. Mass spectra of empty Cp183 particles, in the absence of Impβ, were consistent with the expected populations of capsid morphologies: a majority of T = 4 capsids (5.05 MDa) and a small population of T = 3 capsids (3.83 MDa) (Fig 4A and inset). The CDMS spectra were fit with a series of Gaussian peaks with widths corresponding to the expected uncertainty in the mass measurement. The fits indicate that there is a small high mass shoulder on the main peak extending to 5.7 MDa. The high mass tail is attributed to salt adducts. CDMS spectra that were collected as a function of salt concentration show that the center of the T = 4 peak shifts linearly at a rate of 37.7 kDa per 0.1 M ammonium formate increment. This shift is also attributed to salt adducts and the masses can then be determined by extrapolating to zero salt concentration.

**Fig 5:**
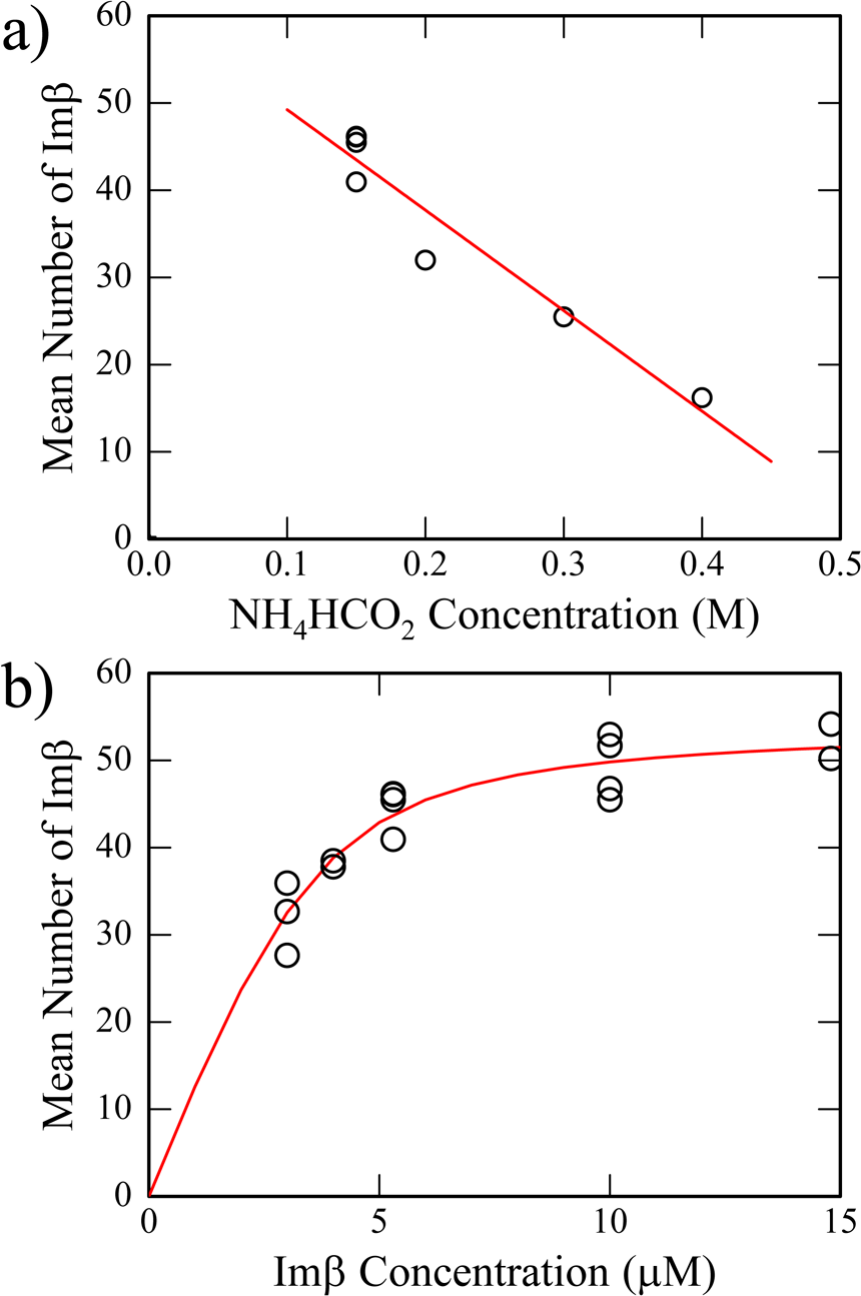
CDMS quantification of Impβ-capsid complexes indicates saturable, ionic strength-sensitive binding of Impβ. (a) The mean number of Impβ bound to Cp183 capsids decreases as a function of NH_4_HCO_2_ concentration. The Impβ concentration was 5.3 μM and the concentration of Cp183 was (7.9 μM dimer, 66 nM capsid). (b) The average number of Impβ bound to HBV Cp183 capsids increases as a function of Impβ concentration. All samples used in this titration were in 150 mM ammonium formate. The binding isotherm fit to these data suggests that up to 50 Impβ bind per capsid with a dissociation constant of about 2.5 μM.

SEC experiments showed binding was sensitive to ionic strength which is indicative of an electrostatic interaction between Impβ and capsids. We observed a similar effect using CDMS to measure the average masses of complexes formed with 66 nM capsid (7.9 μM Cp183 dimer) and 5.3 μM Impβ where the ammonium formate buffer varied from 0.15 to 0.4 M (Fig 5A). The average masses of the complexes decreased linearly with ionic strength. For further CDMS experiments we used 0.15 M ammonium formate, the minimum salt concentration that prevented capsid aggregation and precipitation in the presence and absence of Impβ.

A titration of Cp183 by Impβ was measured by CDMS (Fig 5B). Assuming binding occurred exclusively at the quasi-sixfold axis, we anticipated a maximum mass of 8.04 MDa corresponding to 30 Impβ molecules. In fact, we saw no evidence for accumulation of this complex in mass spectra. This is demonstrated in example spectra where the ratios of Impβ to quasi-sixfold sites were 1.5:1 and 7.5:1 (3.0 and 14.8 μM Impβ with 7.9 μM Cp183 dimer, respectively) (Fig 5A). For the lower ratio, the major peak was very broad and centered at 8.5 MDa, corresponding to T = 4 capsids with ~35 Impβ. At the 7.5:1 ratio, the peak in the mass histogram shifted to 10.3 MDa, which corresponds to a T = 4 capsid with 53 Impβ. The mass distributions with Impβ were very broad, indicating a distribution of Impβ on capsids. For the 7.5:1 ratio, the mass range extended from 7 to 14.5 MDa, corresponding to a range of 20 to 96 Impβ per capsid.

Plotting the average mass of Cp183-Impβ complexes as a function of Impβ concentration, allowed calculation of an apparent dissociation constant based on a fit for non-cooperative binding of many equivalent sites [47]. A least squares fit (the red trace in Fig 5B) returned parameters of 53 sites per capsid each with a K_D_ of 0.7 μM. This K_D_ is notably below the cytosolic concentration of Impβ of 3 to 5 μM [48,49]. However, our CDMS spectra indicate that binding does not saturate at the predicted value, indicating that this fit outlines a much more complex reaction and does not portray a simple binding isotherm. At the highest concentration of Impβ tested, we observed abroad distribution of masses extending to 14.5 MDa (a T = 4 capsid with 96 Impβ) (Fig 4A). It is hard to imagine how 53 Impβ molecules, let alone 96, can bind to equivalent sites on a T = 4 capsid surface, thus these complexes were examined in greater detail.

CDMS provided further information on the homogeneity, or lack thereof, of molecular species by allowing examination of the mass and charge of each ion (Fig 4B). In electrospray, large ions are believed to be generated by a charge residue mechanism [50,51], where the charge on each ion corresponds to the Rayleigh limit for a water droplet with the same diameter [52]. This model has been shown to account for the charges on globular proteins and protein complexes [53,54]. For Cp183 without Impβ, ions fall into two tight groups, *T* = 3 and *T* = 4 capsids, that fall close to the predictions for corresponding molecular and droplet diameters (Fig 4B). The narrow charge distributions found for the *T* = 3 and *T* = 4 capsids are characteristic of an object with a well-defined structure. However, the complexes of Cp183 with Impβ have a very broad charge distribution centered at a much higher charge than predicted by the model. The broad distribution of charges suggests a heterogeneous mixture including non-globular structures. For monomeric proteins, a large charge is usually associated with unfolding to a more extended structure [55], By analogy, to accommodate the number of Impβ adducts and charge, the results shown here suggest that some of the capsids may have broken open.

### Impβ can dissociate capsids or be internalized by them

**Fig 6:**
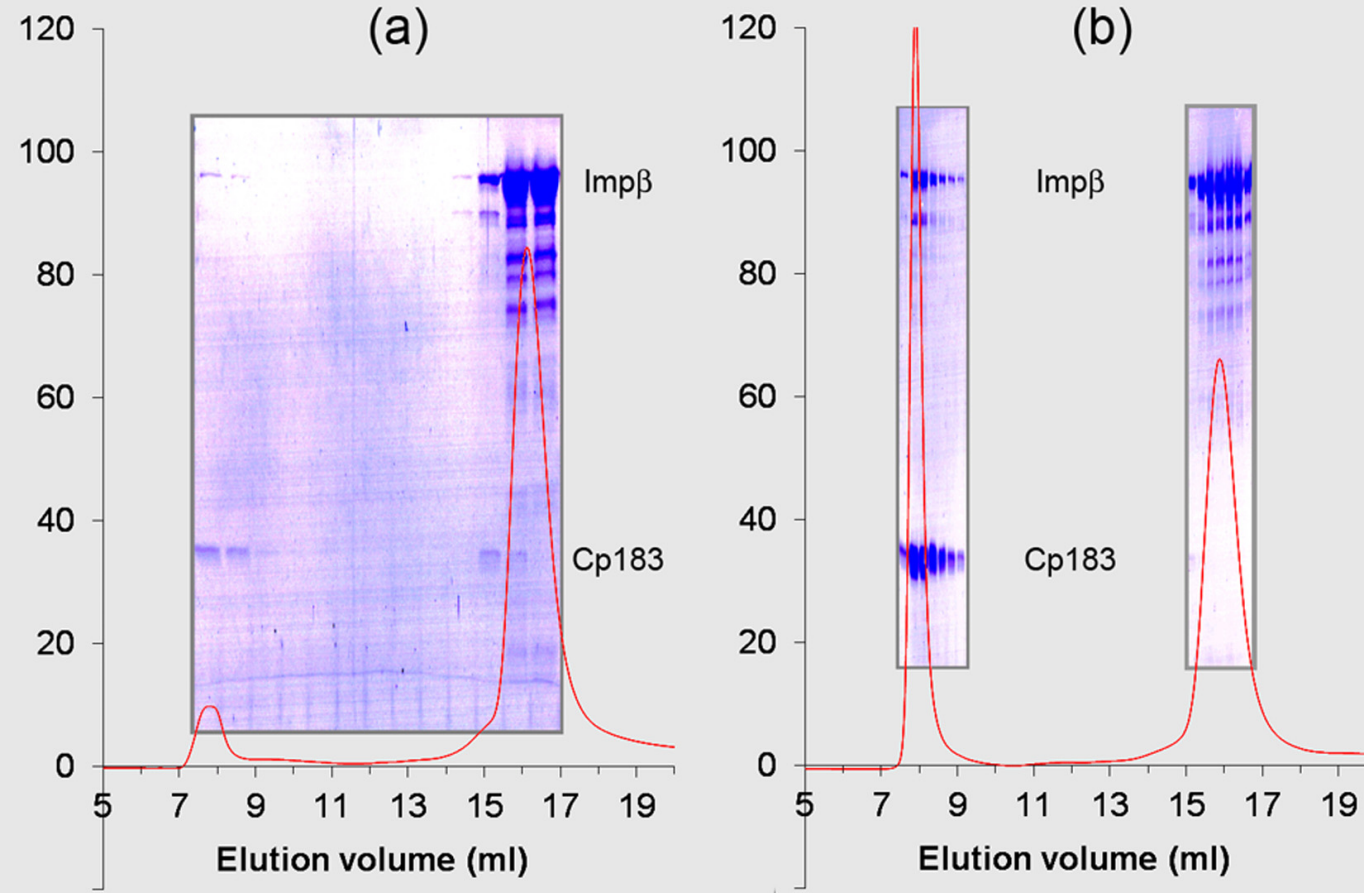
High concentrations of Impβ can lead to dissociation of HBV capsids. The fraction of Cp183 co-eluting with Impβ varies depending on the reaction conditions and the state of the Cp183. (a) 11μM Cp183 capsids with 18.8 μM Impβ in 0.15 M ammonium formate, (b) 11 μM Cp183 in 18.8 μM Impβ in 0.15 M NaCl, 20 mM TrisHCl pH7.4. Images of Coomassie-stained SDS-PAGE of fractions have been aligned with the chromatographs, compressing the images horizontally. The high concentrations of Impβ in these gels shows impurities that are actually present at very low concentrations as shown in Fig 2. Chromatography conditions were the same as in Fig 2.

Initial structural studies suggested the presence of 30 equivalent sites while CDMS titrations provided confounding data that capsids were heterogeneous and could bind more than 90 Impβ molecules, possibly damaging the capsids in the process. To address this conflicting information we examined the effect of excess Impβ on HBV capsid integrity using SEC. We observed three conditions where some Cp183 eluted substantively later than the capsid at ~7.8 ml and earlier than dimer at ~17 ml, co-eluting with the leading edge of the Impβ peak, in fractions between 15 ml and 16 ml:

1. 11 μM Cp183 dimer with 18.8 μM Impβ in 0.15 M ammonium formate at pH 7.4 (205 Impβ:capsid) (Fig 6A),
2. 11 μM Cp183 dimer with 18.8 μM Impβ in 0.15 M NaCl, pH 7.4 (205 Impβ:capsid) (Fig 6B)
3. 7.9 μM Cp183 dimer with 5.3 μM Impβ in 0.15 M NaCl, pH 7.4 (80 Impβ:capsid) (Fig 6C).

Cp183 that co-elutes with Impβ is much smaller than capsids, but larger than Cp183 dimers, and larger than free Impβ, which elutes at 16 ml. Therefore this Cp183 dimer was likely to be part of a complex that included one or two copies of Impβ. We reasoned that the material in the peak was scavenged from capsids dissociated by interaction with excess Impβ.

Reactions where there was a substantial excess of Impβ and relatively low ionic strength provided evidence of capsid dissociation. In contrast, features that attenuated binding and stabilized capsids, e.g. phosphorylated Cp183 (Fig 2D) or higher ionic strength (Fig 2B) prevented measurable dissociation.

### Cryo-EM reconstructions of high Impβ:Cp183 indicate that Impβ may be internalized

**Fig 7:**
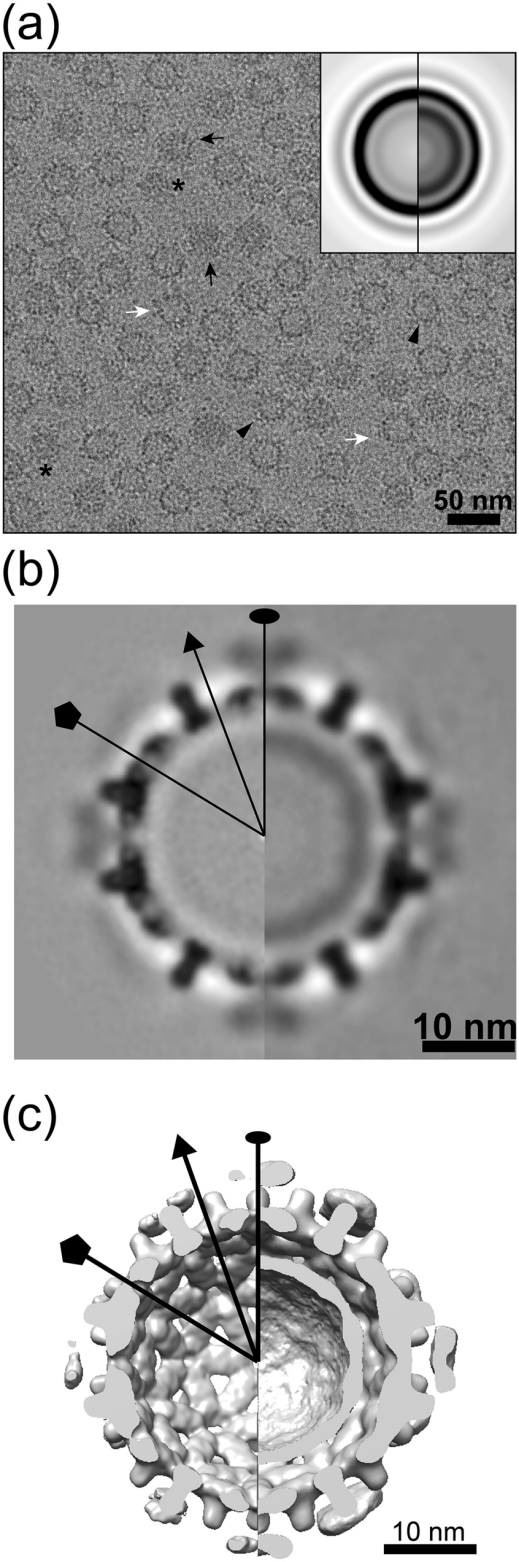
Cryo-EM reconstructions of Cp183-Impβ complex in ammonium formate. The sample was prepared with 11 μM dimer concentration for Cp183 capsid and 18.8 μM Impβ, a ratio of 205 Impβ per capsid. The sample was dissolved in 0.15 M ammonium formate buffer. (a) Typical cryo-EM micrograph of Cp183-Impβ shows morphologically distinct particles: normal particles (white arrow), dark particles (black arrow), T = 3 particles (black arrowhead), and defective particles that include a mixture of fragments (star). The inset shows the translationally and rotationally aligned averaged images: averaged from all 12,004 particles (left) and averaged from 4,516 “dark” particles (right). (b) Central sections and (c) inner surfaces of the 3D reconstructions computed from all particles (left) and dark particles (right). An additional ring of density was observed inside the dark particle. Representative twofold, threefold and fivefold axes of symmetry are indicated by oval, triangle, and pentagon. In (c) the surface shaded interior maps were rendered at the contour levels where the density of Cp183 spike can fully cover the atomic structure. The all-particle map is at a resolution of 10.1 Å while the dark particle map resolution is 13.8 Å (S2 Table).

To comprehend how > 50 Impβ molecules arrange on a capsid, as observed by CDMS in contrast to our expectation of 30 binding sites per capsid, we examined samples with a high Impβ:capsid ratio. Cp183 capsids at 11 μM dimer were incubated with 18.8 μM Impβ in 0.15 M ammonium formate at pH 7.4. Negative stain micrographs of this sample demonstrated a population of typical HBV capsids and a population that appeared to sequester interior stain, “dark particles”. Similarly, cryo-micrographs showed four types of capsid-like particles (Fig 7A): (i) morphologically normal capsids (Fig 7A, white arrow), (ii) a minor population of T = 3 capsids (Fig 7A, black arrowhead), (iii) defective particles suggestive of capsids caught in the act of dissociation (Fig 7A, star), and (iv) “dark particles” that have an exterior boundary that is indistinguishable from normal capsids but a dark interior suggestive of internal content (Fig 7A, black arrow, see also S4 Fig). Confirmatory reference free classification showed three different major types: 1) empty 30nm (T = 3) capsids decorated with Impβ, 2) empty 36nm (T = 4) capsids decorated with Impβ, and 3) 36nm (T = 4) capsids decorated with Impβ with an additional inner ring (S4 Fig); with the last type corresponding to dark particles.

Initially, we pursued cryo-EM image analysis of all T = 4 particles combined, determining a 3D structure to 10.1 Å resolution (Fig 7B and 7C, left half; S1 Table). The outer surface of the cryo-EM density map was very similar to reconstructions of low Impβ:capsid ratio complexes (Fig 3). Impβ density was observed at the 30 quasi-sixfold axes (Fig 7B and 7C, left half). However, we also observed a weak internal layer of density in 2D averages of the raw images (Fig 7A, inset, left half) and in 3D reconstructions (Fig 7B, left half).

Dark particles were then manually separated from the other ~36nm particles for structural elucidation. A translationally aligned, averaged image showed an inner annulus of density with an outer radius of ~12 nm and a width of ~4 nm (Fig 7A, inset, right). Because the sample had no nucleic acid content, based on the UV absorbance, and the location of the internal density is different from that of encapsidated RNA [13], we exclude the possibility of nucleic acid contamination during sample preparation.

**Fig 8:**
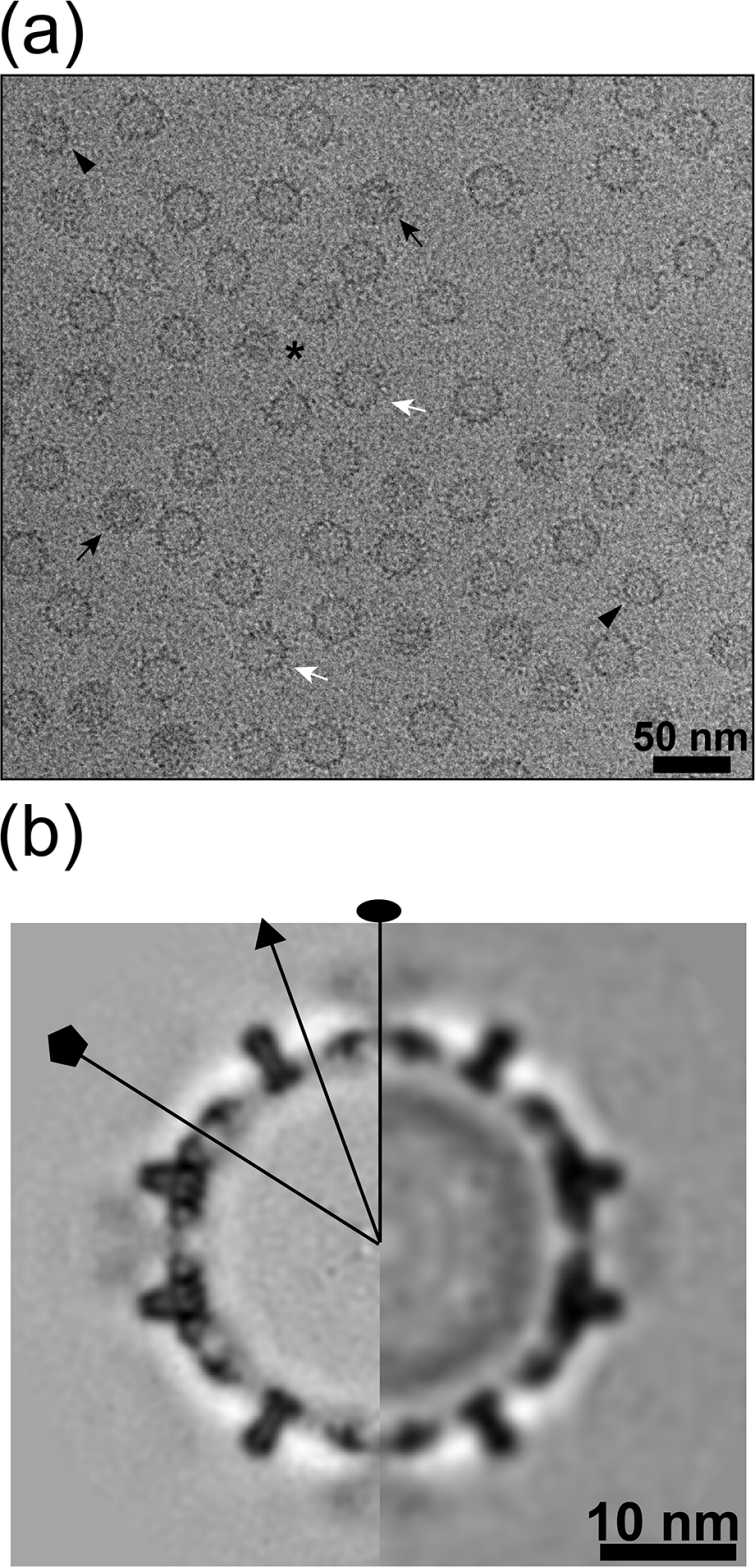
Cryo-EM reconstruction of Cp183-Impβ complex in sodium chloride. (a) Representative cryo-EM micrograph of Cp183 capsid (11 μM dimer) and 18.8 μM Impβ, a ratio of 205 importins per capsid, in 0.15 M NaCl. Most particles appear to be empty (white arrow), but some dark particles (black arrow), T = 3 particles (black arrowhead), and defective particles (star) were observed. (b) The central sections of the 3D reconstructions, computed from all particles (left) and the dark particles (right), show an internal dark ring of density. Oval, triangle, and pentagon indicate locations of twofold, threefold and fivefold axes, respectively. The reconstructions were determined to 8.9 Å resolution for all particles and 15.9 Å resolution for the dark particles.

An image reconstruction of the dark particles was calculated to 13.8 Å resolution (Figs 7B and 7C and S5). We found a typical T = 4 HBV capsid with Impβ density at quasi-sixfolds and inside the capsid we observed a novel sphere of density. The capsid shell, after adjusting magnification, fit a 1QGT molecular model without modification (S5 Fig). The external Impβ density at each quasi-sixfold was consistent with an Impβ atomic structure (PDB entry [3LWW]) that was circularly averaged about the respective axes. As with reconstructions at lower Impβ concentration, there was no evidence of Impβ density on the capsid fivefolds. The internal sphere of density could not be interpreted at a molecular level and was not stronger under fivefold or quasi-sixfold vertices, where CTDs are clustered. To ensure that dark particles were not a function of the ammonium formate buffers used in the experiment and CDMS, we obtained similar cryo-EM data for samples with more typical buffer conditions (Fig 8, 11 μM Cp183 dimer with 18.8 μM Impβ in 0.15 M NaCl, pH 7.4).

Given the CDMS data, it is likely that that dark particles represent capsids with a broad distribution of internalized Impβ molecules. If the internal mass was contributed by Impβ, the volume of the density (between radii of 83 and 122 Å) would accommodate a maximum of 48 closely packed Impβ molecules (assuming a protein density of 1.43 g/cm^3^), resulting in particles with up to 78 Impβ molecules. Furthermore, it is attractive to speculate that the broken and compromised particles observed in micrographs, relatively unusual for empty capsids, could be associated with very large numbers of importins.

### Importin β, but not importin α, binds to unassembled HBV core protein-GST fusions

Nuclear transport of mature cores uses an Impα+Impβ complex yet in this study we show binding of empty HBV cores by Impβ. This led us to question the interaction of importins with unassembled core protein dimers. Because of the limited solubility of Cp183 in vitro, we adopted a different assay strategy.

IBBs consist of multiple basic amino acids scattered over a 39 amino acid segment of protein [56]. This implies that Impβ binding to the empty capsids is based on exposure of the entire 34-residue CTD plus part of the assembly domain, which is supported by recent studies showing that trypsin cleavage of Cp183 capsids can involve CTD exposure beyond residue 150 [17]. To remove any ambiguity regarding the assembly state, we examined Impβ interaction with Cp expressed as a fusion protein with a GST (Glutathione S-transferase) at its N terminus, which interferes with capsid formation [57]. To directly demonstrate Impα and Impβ interaction with GST-Cp183, we used a bead halo assay [58], where sepharose beads coated with GST-Cp183 act as bait for fluorescent Impα and Impβ. Having confirmed GST (and GST-Cp183) bound to the beads by Western blot, we quantified importin binding by confocal laser scan microscopy in the focal planes of the beads.

**Fig 9:**
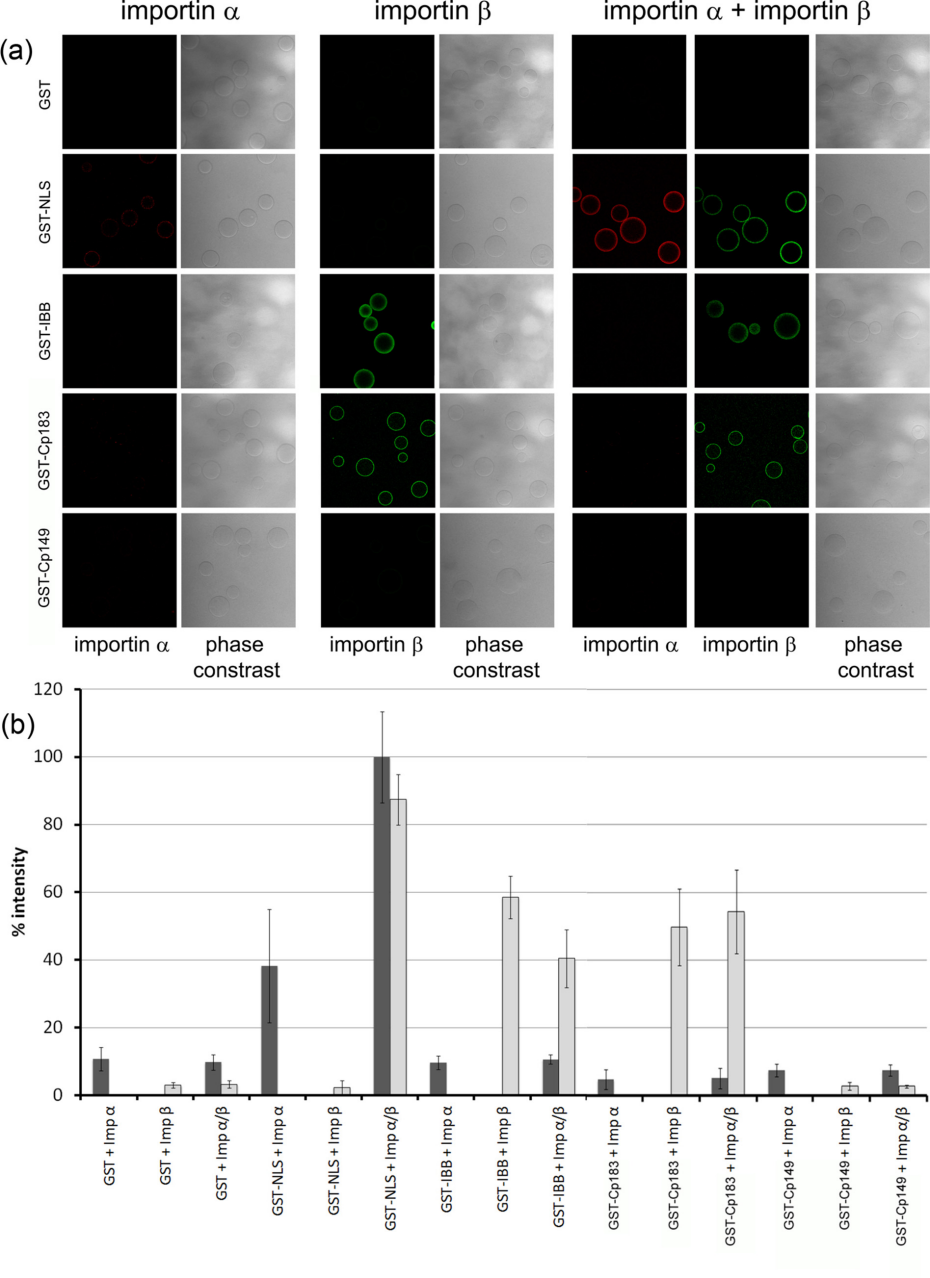
Importin β interaction with GST-Cp183 fusion protein. (a) Confocal fluorescence microscopy of importin α-Alexa594 and importin β-Alexa488 with glutathione sepharose, coated with different GST fusion proteins. The protein coating the beads is indicated on the left. The importins in the binding reaction are shown at the top of the figure; the read out channel on the bottom. (b) Quantification of the fluorescence. Importin α: dark grey bars, importin β: light grey bars. The bars give the average of three independent experiments; the ranges are indicated. Y axis: % relative to the mean of importin β binding to GST-NLS; x axis: upper row–importin in the binding reaction, lower row–substrate on the beads.

As shown in Fig 9, neither Impα nor Impβ bound to beads coated with GST alone. GST fused to the prototype NLS of SV40TAg interacted weakly with Impα alone but much stronger with Impα+Impβ. This finding is in agreement with Falces et al. [59] showing that Impβ enhances binding of Impα to the NLS of nucleoplasmin by nine-fold. A GST-IBB fusion protein bound Impβ directly but failed to interact with Impα alone. Addition of an Impα+Impβ mixture neither increased binding of Impα nor decreased binding of Impβ. GST-Cp183 coated beads exhibited a strong Impβ binding in the absence and presence of Impα but did not detectably bind Impα. GST-Cp183 thus showed the same binding pattern as GST-IBB. This suggests a difference in interaction of Impα+Impβ with unassembled Cp183 versus assembled capsid that bears more complete investigation. The Impβ interaction was dependent upon the CTD as a CTD deletion mutant also fused to GST, GST-Cp149, did not interact with either importin.

## Discussion

In an infection or HBV expression system, core protein is overexpressed with respect to the amount needed for virion formation. Some of the resulting empty capsids are secreted from the host cell. As much as 99% of enveloped cores are empty [1,60]. In most infected patients, most core protein is localized to the nucleus [32–34]. Most nuclear core protein is unlikely to be derived from infecting HBV since only a few virions are taken up and hepatocytes are resistant to new infections after infection is established on the cellular level. The interaction of empty cores with nuclear transport proteins has been an open question.

Mature HBV cores depend on Impα and Impβ for nuclear transport [8]. In this paper we observe that empty cores and free Cp183 bind to Impβ without the Impα adapter (Figs 2 and 9). HBV capsids undergo a structural changes upon genome maturation associated with capsid destabilization [61] and exposure of the last parts of the CTD. In mature capsids, this leads to Impα mediated interaction with Impβ [8]. Normally, immature RNA-filled and ssDNA-filled cores do not bind importins, though phosphorylation can modulate transient CTD exposure in RNA-filled cores presumably by attenuating interaction with nucleic acid [8,17]. For empty capsids we found that Impα was dispensable for Impβ binding. Though empty capsids could bind Impα+Impβ, unassembled GST-Cp183 exclusively bound Impβ. This implies a structural difference in the CTD presentation in empty and DNA-filled mature capsids. We propose that in empty capsids a larger fraction of the CTD is available than in nucleic acid-filled capsids, exposing the several arginine clusters needed to comprise an IBB. Thus, both Impα+Impβ and Impβ import paths have plausible but distinct roles in the HBV lifecycle.

In addition to direct interaction of Impβ with empty capsids, two observations are peculiarly striking in this study: Impβ can destabilize capsids and Impβ can infiltrate HBV capsids. Apparently, Impβ provides an additional destabilizing influence, perhaps due to mechanical crowding at the quasi-sixfold vertex or by requiring interaction with a more extended segment of the CTD. Cryo-EM reconstructions showed that Impβ on the exterior of capsids is localized to the thirty quasi-sixfold vertices (Figs 3, 7 and 8). The quasi-sixfold vertices have a hole large enough to allow CTDs to extrude from the capsid [16,17] and space to fit one Impβ. However titrations of empty capsid by Impβ and single molecule mass measurements by CDMS showed that as many as 96 Impβ molecules could be bound in a Cp183 complex, far more Impβ than could reasonably decorate the exterior of the capsid. Though in a different environment, similar infiltration of proteins into a closed capsid have been observed artificial capsids [62]. Broken capsids and dark particles together provide a basis for explaining the presence of more than 30 Impβ molecules per capsid, though the evidence is circumstantial. In cryo-EM of capsid samples prepared with high Impβ concentrations, we observed that the sample was enriched with Impβ only in broken particles and “dark particles”. On reconstruction, it was apparent that dark particles contained considerable internal density; the only sources for this density were Impβ or Cp183 that had been released from disrupted capsids. These particles are heterogeneous (Fig 4A) and those with the highest Impβ are likely to be broken, though only a small fraction, <5% of Cp183, was dissociated to Impβ-associated dimer (Fig 6). Under the conditions tested, empty Cp183 capsids are at the edge of their stability under these solution conditions [63]. Capsid fragility and the intrinsic dynamic behavior of capsids, based on H-D exchange experiments [64], are consistent with the hypothesis that capsids breathe and can transiently open to “swallow” bound Impβ.

Cytoplasmic capsid destabilization may have important biological roles: (i) exposure of the contents of infecting capsids to facilitate partial deproteinization of the rcDNA (i.e. proteolysis of the packaged reverse transcriptase) [15], (ii) dissociation of empty capsids and (iii) transport of dimers and/or intact empty capsids to nuclear pores. Nuclear core protein is associated with virus function including binding to viral nucleic acid [65–67]. Due to the preponderance of Impβ in the cytoplasm, Impβ-mediated destabilization of empty capsids should occur exclusively in this compartment, which is thus consistent with the accumulation of empty capsids in the nucleus [1,61,68].

The intracellular roles of empty cores are essentially unexplored. We speculate that empty capsids could be a storage pool for core protein bound for the nucleus and for construction of new virus, analogous to the role proposed for empty picornavirus capsids [69–71]; in this scenario, secretion of empty capsids would be homeostatic. Accumulation of capsids may modulate the interaction between infected cell and the immune system. We speculate that if HBV capsids were allowed to accumulate in the cytoplasm they could become subject to proteasomal degradation. This is seen with some core protein-directed antivirals [38]. Entry of the proteolytic fragments into the MHC class I pathway could then proceed via the ER and Golgi-bound TAP (transporter associated with antigen processing), leading to core epitope exposure on the surface of hepatocytes. This hypothesis is in agreement with the observation that cytosolic capsids are associated with liver inflammation in infected patients [35,36]. The degree of hepatocyte cytoplasmic expression of hepatitis B core antigen correlates with the histologic activity of liver disease in the young patients with chronic hepatitis B infection [36,37]. Though a mechanistic connection has not been made, detection of hepatitis B virus antigens in liver tissue has been related to viral replication and histology in chronic hepatitis B infection [37]. Thus, a second path for removal of empty capsids could be important for establishing and maintaining chronic HBV infection.

We must note the importance of single molecule techniques for analyzing Cp183 interaction with Impβ. Cryo-EM allowed visual examination of individual particles and their subsequent classification. CDMS revealed a far richer level of detail than would have been possible with bulk measurements. The ability to determine mass for each ion allows investigation of complex mixtures. Without CDMS, the titration analysis (Fig 5) would have depended on an average ratio from a bulk technique (e.g. SDS-PAGE analysis of SEC data), missing the heterogeneity, including the unexpectedly large numbers of Impβ for some core protein complexes. The distribution of charge states further indicated a structural flexibility and heterogeneity not seen in capsids without Impβ (Fig 4B). This specific observation led to closer examination of capsid structure and provides insight into capsid dynamics.

The fundamental observation of this paper is that HBV core protein dimer and empty HBV capsids interact with the Impβ. The recent observations of the prevalence of empty capsids in chronic infection [1,60] emphasizes the importance of understanding cellular responses to such capsids. From a physical perspective, the response of the capsid to Impβ offers new clues to capsid dynamics. Impβ entry into a capsid seems unlikely to proceed by complete dissociation and reassembly: if dissociation of Cp183 with two bound Impβ were thermodynamically favored we would expect that high concentrations of Impβ would exclusively lead to Impβ-decorated dimer. As complete dissociation was not observed, even after up to two days of incubation, we are led to the hypothesis that excess Impβ may decrease the energy barrier to transiently opening a capsid. The biological implications of accumulation of empty capsids, discussed above, range from distinct intracellular trafficking paths to immune modulation. In summary, further investigation of the interaction of empty, RNA-filled, and DNA-filled capsids with Impβ and/or Impα will provide additional insight into the intracellular trafficking.

## Methods

### Preparation of Cp183, phosphorylated Cp183 (P-Cp183) and Cp149

Cp183, P-Cp183 and Cp149 dimer and capsids were prepared as previously described [16,43,72,73]. Concentrations of dimer and empty capsids were quantified by absorbance using a per dimer extinction coefficient of ε_280_ = 60,900 M^-1^cm^-1^)[43]. For Cp183 or P-Cp183 capsids incorporating *E*. *Coli* RNA, the protein concentration was determined by SDS-PAGE and compared to a standard curve. The resulting protein had a calculated mass of 21.0 kDa.

### Preparation of His-tagged human importin β1 (Impβ)

The Impβ clone was a kind gift of Dr. Lane Baker. His-tagged Impβ was expressed from a PET30a vector [74]. Lysate from IPTG-induced cells was loaded onto a His-Trap column, washed with at least 10 column volumes of 20 mM imidazole + 0.5 M NaCl at pH 7.4 and eluted with 0.5 M imidazole + 0.5 M NaCl at pH 7.4. Peak fractions were desalted/dialyzed into 0.15 M NaCl 20mM Tris pH 7.4 and loaded onto a HiTrap SP HP column to which only contaminants bound. The Impβ was then loaded onto a HiTrap Q FF column and eluted by 2 M NaCl 20mM Tris pH 7.4. The concentrated protein solution was dialyzed against 0.15 M NaCl, 10 mM DTT, 20 mM Tris-HCl at pH 7.4, for storage at -20°C. Prior to binding experiments, Impβ stock solutions were further purified through a Superose 6 column with the running buffer 0.5 M NaCl, 10 mM DTT, 20 mM Tris-HCl at pH 7.4 (S1 Fig). The resulting protein had a calculated mass of 97.0 kDa.

### Capsid-Impβ binding assays

To test for Impβ-binding, capsid samples were dialyzed into 0.5 M NaCl, 10 mM DTT, 20 mM Tris-HCl pH 7.4, and then mixed with Impβ in the same buffer. The mixture was dialyzed overnight against 0.15 or 0.25 M NaCl, 10 mM DTT, 20 mM Tris-HCl pH 7.4. CDMS samples were first dialyzed against 0.15 M NaCl, 10 mM DTT, 20 mM Tris-HCl pH 7.4 overnight, and then against 0.15 M ammonium formate pH 7.4 for a second day. For column assays, 150 μl samples were run through a Superose 6 column equilibrated in the final dialysis buffer at 0.5 ml/min. Throughout this process, all samples were kept at 4°C. In control experiments to identify the elution volume of free dimer, the running buffer included a non-denaturing 1.5 M guanidine hydrochloride not present in the experiments with Impβ [43].

### cryo-EM of capsid-Impβ complexes

For cryo-EM, Impβ-capsid complexes were prepared with initial concentrations of 7.9 μM Cp183 dimer (in capsid form) with 5.3 μM Impβ or 11 μM Cp183 dimer (in capsid form) with 18.8 μM Impβ. These complexes were assembled by overnight dialysis into 0.15 M NaCl, 10 mM DTT, 20 mM Tris-HCl at pH 7.4. In some cases this was followed by dialysis into 0.15 M ammonium formate pH 7.4. Samples were then concentrated by Nanosep 100K centrifugal device (Pall) to the suitable concentration for cryo-EM.

All cryo-EM data were collected with a JEM-3200FS electron microscopy (JEOL) operated at 320 kV. Specimen preparation and EM operation followed procedures described elsewhere [13]. Briefly, a 4 μl drop of sample solution was applied to a glow-discharged holey carbon grid (Quantifoil R2/2) or continuous carbon film coated grid (EMS). The grids were vitrified in liquid ethane using a FEI Vitrobot. The sample preparation condition was set at 6°C, 100% humidity, and 4 s blotting time. Cryo-EM images were acquired using a Gatan Ultrascan 4000 CCD camera at the nominal magnifications of 40,000x or 80,000x, which are equal to 2.94 Å and 1.48 Å per pixel, respectively. The slit width for the energy filter was set at 20 eV. Detailed information for each dataset is listed in S1 Table.

Image processing and 3D reconstructions were performed by the single particle approach with EMAN2 and AUTO3DEM software packages [75,76]. The quality and the defocus value of each micrograph were determined using CTFFIND3 [77]. Micrographs with significant drift or astigmatism were discarded. Particle images, representing different orientations in the 3D space, were boxed out from the micrograph using e2boxer.py. The images were then normalized and the initial model was built de novo for each dataset [78]. The initial model was then refined by the iterative process of alignment and icosahedral averaging. During processing, only phase information was applied to the contrast transfer function. This refinement process continued until no further improvement was achieved in the 3D model. The resolution of the final reconstruction was estimated based on a Fourier shell correlation (FSC) at 0.5 (S6 Fig). The 3D reconstruction of dark particles prepared from Cp183 (11 μM dimer) and Impβ (18.8 μM) in NaCl was estimated using gold-standard FSC at 0.143. The 3D reconstructions were visualized by RobEM and UCSF Chimera [79].

The molecular modeling analyses was performed by fitting the known atomic structures of HBV (PDB code: 1QGT) and Impβ (PDB code: 3LWW) into the cryo-EM density map using UCSF Chimera.

The cryo-EM electron density maps have been deposited to EMDataBank.org. The accession numbers are EMD-3266, EMD-3267, EMD-3268, EMD-3269, EMD-3270 and EMD 3271, respectively (S2 Table).

### Charge detection mass spectrometry

Sample preparation was the same as described for cryo-EM.

In charge detection mass spectrometry (CDMS) the *m*/*z* and *z* of each ion are measured simultaneously. This allows the masses to be determined for complex mixtures of large ions that are not amenable to study by conventional mass spectrometry, where only the *m*/*z* is measured. The home-built charge detection mass spectrometer used in this study is described in detail elsewhere [80,81]. Briefly, ions are generated by nanoelectrospray and introduced into the instrument through a heated, stainless-steel capillary. The ions are first separated from the ambient gas flow by three differentially pumped regions containing RF ion guides. They are then accelerated to a kinetic energy of 100 eV/charge and focused into a dual hemispherical deflection analyzer that selects ions with a narrow band of kinetic energies. The energy-selected ions then pass into a modified cone trap, which contains the charge detection tube at its center. Trapped ions oscillate back and forth through the tube. When in the tube, the ion induces a charge which is detected by a charge-sensitive preamplifier. The output from the preamplifier is digitized and sent to a computer for analysis using a fast Fourier transform. The *m*/*z* of the trapped ion is determined from the frequency of the fundamental and the charge, *z*, is related to the magnitude of the fundamental. Multiplying *m*/*z* and *z* for each ion gives the mass. The masses are then binned to form a mass histogram. Only ions trapped for the entire trapping period (~94 ms) are included.

The HBV capsids are assembled in NaCl. Nonvolatile salts like NaCl suppress the ion signal from electrospray and lead to mass spectra with unresolved features due to extensive adduct formation [82]. A salt concentration greater than around 100mM is needed to maintain the integrity of the HBV capsids, so in order to obtain useful mass spectra it is necessary to replace the nonvolatile salt with a volatile one. Ammonium acetate is usually the salt of choice. However, in this work we found that ammonium formate provided better mass spectra and so after assembly in sodium chloride, the capsids were dialyzed in ammonium formate for the CDMS studies.

The binding model used to analyze the titration of Cp183 by Impβ is based on a macroscopic dissociation constant that changes with the saturation of the target protein:

$$K_{d}\prime (m)=K_{d}\frac{m}{n-m+1}$$

where *K*_*d*_ is the microscopic dissociation constant (i.e., the dissociation constant for a single site), *n* is the number of sites available, and *m* is the number of sites filled.

### Halo assay

*E*. *coli*-expressed GST proteins were purified using GSTrap FF columns (GE Healthcare) and dialyzed against 50 mM Tris HCl pH7.5, 50 mM NaCl, 5% glycerol, 2mM DTT, 250 mM sucrose. Proteins included a full-length core, GST-Cp183, and a C-terminal domain deletion, GST-Cp149, which lacked all 34 residues of the CTD.

*E*. *coli*-expressed importin α and β were purified using HisTrap FF columns (GE Healthcare), and were labeled with Alexa Fluor 488 (or 594) using Microscale Protein Labeling Kits according to the vendor (Invitrogen). Further purification was realized using Zeba spin desalting columns 7K MWCO (Thermo Fisher Scientific) according to the vendor’s instructions.

The GST-proteins were bound to glutathione sepharose beads for 2 h at 4°C while mixing on a rotation wheel. Beads were then subjected to 5 washes in washing buffer (1 x PBS, 500 mM NaCl, 1% (w/v) BSA). Then 2.5μg of importin α or/and 2.5μg importin β were added to the beads and incubated for 2 h at 4°C. The beads were washed 5 times with washing buffer followed by immediate analysis by microscopy using a SP5 Leica confocal microscope, 20 X objective with the standard settings for the respective fluorophore.

## Supporting Information

S1 Fig Coomassie stained SDS-PAGE of Impβ. (PDF)

S2 Fig ESI mass spectra phosphorylated Cp183, P-Cp183, prepared by co-expression of Cp183 and SRPK (a) in capsids incorporating *E*. *Coli* RNA and (b) in empty capsids from purified P-Cp183. The peak labels show assignment, mass, and intensity.
(PDF)

S3 Fig Fitting an atomic model of Impβ into cryo-EM density. (a) The difference map of Impβ (red) was calculated by subtracting P-Cp183 capsid (Fig 3D) from P-Cp183-Impβ complex (Fig 3E). Prior to the subtraction, the sizes of these two maps were scaled and the density corresponding to the capsid shell region was normalized. The resulting Impβ density (red) was superimposed on the P-Cp183 capsid (gray) and the Impβ density was rendered at the contour level that fully covers the atomic structure of Impβ (PDB code 3LWW). Oval, triangle, and pentagon indicate locations of twofold, threefold and fivefold axes, respectively. (b) The enlarged view and the (c) 90°-tilted view at the twofold location. As the Impβ density is heavily averaged and relatively weak, the fits are presented to allow the reader to gauge the amount of space available.
(PDF)

S4 Fig Reference-free 2D classification of Cp183-Impβ in 0.15 M ammonium formate. Samples of high Impβ:capsid complexes (11 μM Cp183 dimer with 18.8 μM Impβ) were examined by cryo-EM. Unbiased 2D classification using RELION [84] showed three types: (i) T = 4 Cp183-Impβ particles, (ii) T = 4 Cp183-Impβ particles with dark interior, an additional ring-like density (red box), and (iii) T = 3 Cp183-Impβ particles (green box).
(PDF)

S5 Fig Fitting atomic models into cryo-EM of the Cp183/Impβ complex (upper panel). The atomic model of HBV (PDB code 1QGT, color in red, green, cyan and yellow) was fitted into cryo-EM density of Cp183/Impβ complex prepared in 0.15M ammonium formate. The left half of each panel shows the 3D reconstruction computed from whole particles and the right half shows the 3D reconstruction computed from the dark particles. Enlarged views show the Impβ (PDB code 3LWW, color in blue) fitted into cryo-EM density map which rendered at the low contour level. Oval, triangle, and pentagon indicate locations of twofold, threefold and fivefold axes, respectively. The lower panel shows the difference map between the 3D reconstruction computed from whole particles (left) and the 3D reconstruction computed from the dark particles (right). This is essentially a featureless hollow sphere. The contour level was chosen to match the internal density within the 3D reconstruction computed with dark particles. To facilitate comparison, both maps were calculated at 15 Å resolution. As the Impβ density is heavily averaged and relatively weak, the fit in the inset is presented to allow the reader to gauge the amount of space available.
(PDF)

S6 Fig Resolution estimation. The black (phosphorylated empty Cp183), green (phosphorylated Cp183 with Impβ) and red (Cp183 with Impβ) curves show the FSC for the 3D reconstructions in Fig 3. The blue curves (thick, all particles; thin, dark particles) show the FSC for the 3D reconstruction in Fig 7. The gray curves (thick, all particles; thin, dark particles) show the FSC for the 3D reconstruction in Fig 8. The 3D reconstruction of the dark particles in NaCl was calculated using gold-standard FSC.
(PDF)

S1 Table Estimated Impβ: Capsid ratio determined from size exclusion chromatography. As SEC is not an equilibrium technique, Impβ can dissociate during the course of the 40 minute-long experiment. In addition, some capsid-Impβ complex may precipitate before or during chromatography. These complications result in an underestimate of capsid-bound Impβ.
(PDF)

S2 Table Data collection information. Data are presented for seven reconstructions of the full-length core protein Cp183 with and without Impβ.
(PDF)
