# Supplementary figures and images for: Importin β Can Bind Hepatitis B Virus Core Protein and Empty Core-Like Particles and Induce Structural Changes
Source: PLoS Pathog. 2016 Aug 12;12(8):e1005802. doi: 10.1371/journal.ppat.1005802 (PMC4982637; doi:10.1371/journal.ppat.1005802)

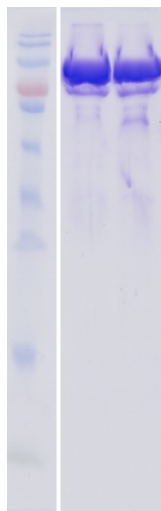

**S1 Figure.** Coomassie stained SDS-PAGE of Imp $\beta$ .

Supplement: S1 Fig — (PDF) [file ppat.1005802.s001.pdf]
