# Supplementary material for: Importin β Can Bind Hepatitis B Virus Core Protein and Empty Core-Like Particles and Induce Structural Changes
Source: PLoS Pathog. 2016 Aug 12;12(8):e1005802. doi: 10.1371/journal.ppat.1005802 (PMC4982637; doi:10.1371/journal.ppat.1005802)
Supplement: S2 Fig — ESI mass spectra phosphorylated Cp183, P-Cp183, prepared by co-expression of Cp183 and SRPK (a) in capsids incorporating E. Coli RNA and (b) in empty capsids from purified P-Cp183. The peak labels show assignment, mass, and intensity. (PDF) [file ppat.1005802.s002.pdf]

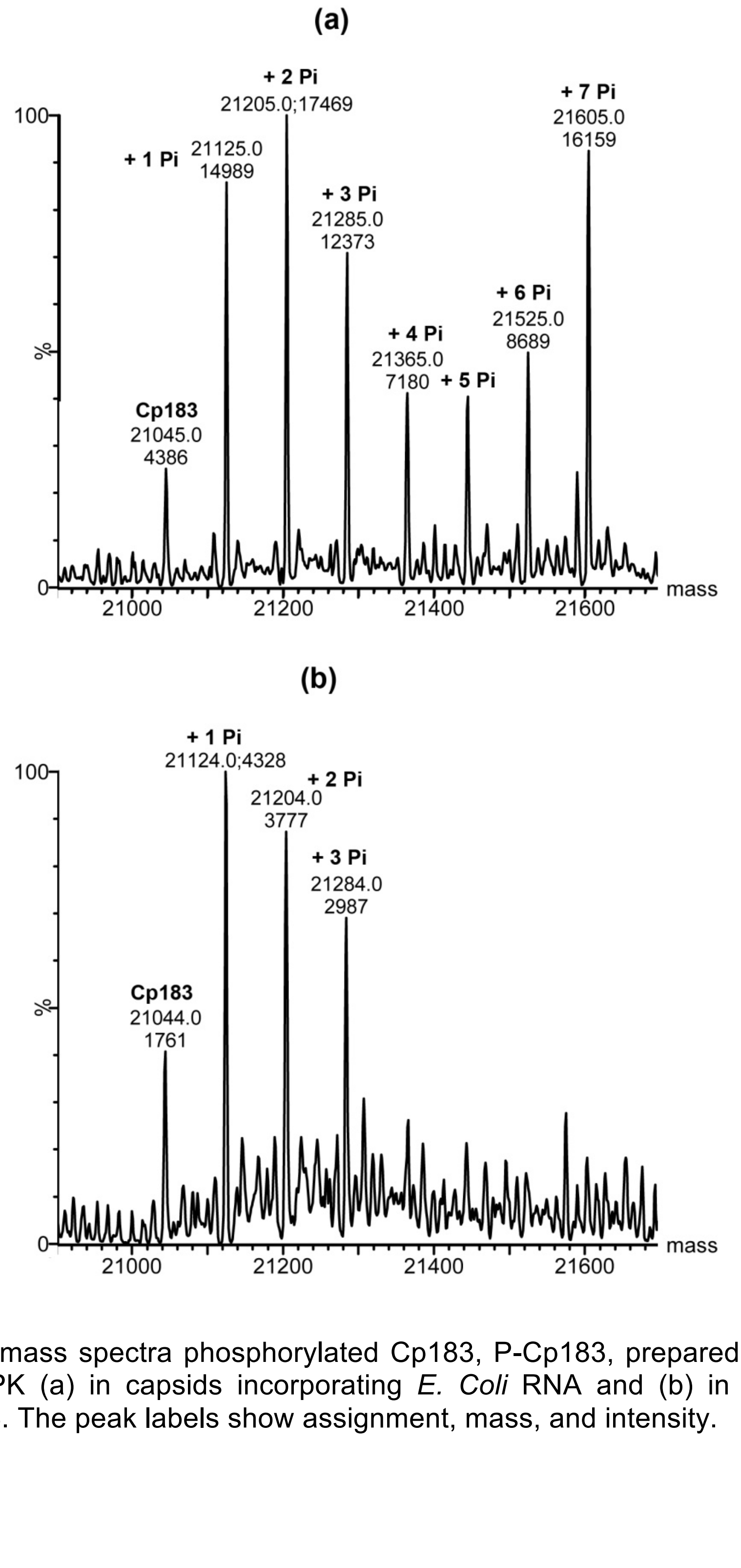

S2 Figure. ESI mass spectra phosphorylated Cp183, P-Cp183, prepared by co-expression of Cp183 and SRPK (a) in capsids incorporating *E. Coli* RNA and (b) in empty capsids from purified P-Cp183. The peak labels show assignment, mass, and intensity.
