# Supplementary material for: Importin β Can Bind Hepatitis B Virus Core Protein and Empty Core-Like Particles and Induce Structural Changes
Source: PLoS Pathog. 2016 Aug 12;12(8):e1005802. doi: 10.1371/journal.ppat.1005802 (PMC4982637; doi:10.1371/journal.ppat.1005802)
Supplement: S3 Fig — (a) The difference map of Impβ (red) was calculated by subtracting P-Cp183 capsid (Fig 3D) from P-Cp183-Impβ complex (Fig 3E). Prior to the subtraction, the sizes of these two maps were scaled and the density corresponding to the capsid shell region was normalized. The resulting Impβ density (red) was superimposed on the P-Cp183 capsid (gray) and the Impβ density was rendered at the contour level that fully covers the atomic structure of Impβ (PDB code 3LWW). Oval, triangle, and pentagon indicate locations of twofold, threefold and fivefold axes, respectively. (b) The enlarged view and the (c) 90°-tilted view at the twofold location. As the Impβ density is heavily averaged and relatively weak, the fits are presented to allow the reader to gauge the amount of space available. (PDF) [file ppat.1005802.s003.pdf]

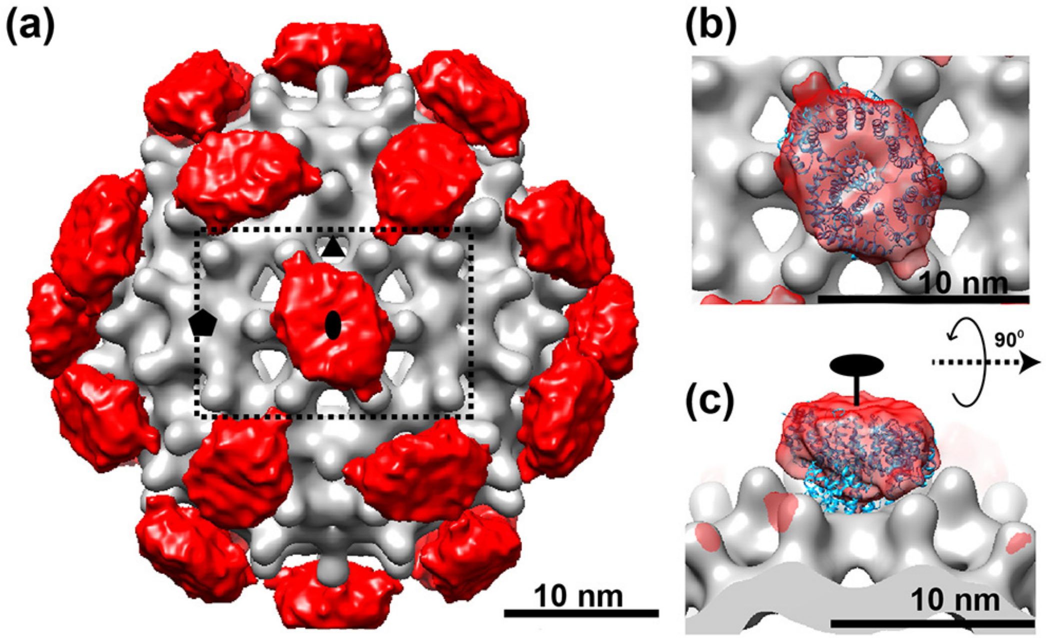

S3 Figure. Fitting an atomic model of Impβ into cryo-EM density. (a) The difference map of Impβ (red) was calculated by subtracting P-Cp183 capsid (Fig. 3d) from P-Cp183-Impβ complex (Fig. 3e). Prior to the subtraction, the sizes of these two maps were scaled and the density corresponding to the capsid shell region was normalized. The resulting Impβ density (red) was superimposed on the P-Cp183 capsid (gray) and the Impβ density was rendered at the contour level that fully covers the atomic structure of Impβ (PDB code 3LWW). Oval, triangle, and pentagon indicate locations of twofold, threefold and fivefold axes, respectively. (b) The enlarged view and the (c) 90°-tilted view at the twofold location. As the Impβ density is heavily averaged and relatively weak, the fits are presented to allow the reader to gauge the amount of space available.
