# Supplementary material for: Importin β Can Bind Hepatitis B Virus Core Protein and Empty Core-Like Particles and Induce Structural Changes
Source: PLoS Pathog. 2016 Aug 12;12(8):e1005802. doi: 10.1371/journal.ppat.1005802 (PMC4982637; doi:10.1371/journal.ppat.1005802)
Supplement: S4 Fig — Samples of high Impβ:capsid complexes (11 μM Cp183 dimer with 18.8 μM Impβ) were examined by cryo-EM. Unbiased 2D classification using RELION [84] showed three types: (i) T = 4 Cp183-Impβ particles, (ii) T = 4 Cp183-Impβ particles with dark interior, an additional ring-like density (red box), and (iii) T = 3 Cp183-Impβ particles (green box). (PDF) [file ppat.1005802.s004.pdf]

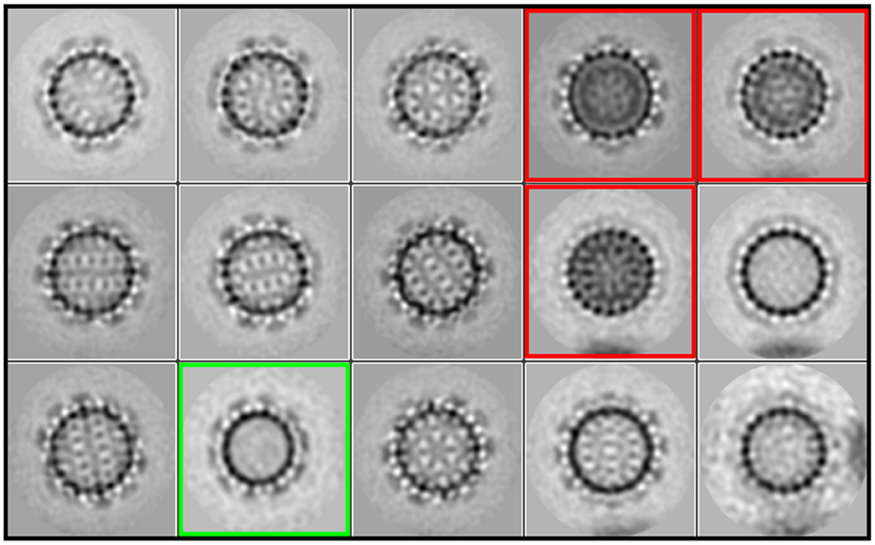

S4 Figure. Reference-free 2D classification of Cp183-Impβ in 0.15 M ammonium formate. Samples of high Impβ:capsid complexes (11 μM Cp183 dimer with 18.8 μM Impβ) were examined by cryo-EM. Unbiased 2D classification using Relion [84] showed three types: (i) T=4 Cp183-Impβ particles, (ii) T=4 Cp183-Impβ particles with dark interior, an additional ring-like density (red box), and (iii) T=3 Cp183-Impβ particles (green box).
