# Supplementary material for: Importin β Can Bind Hepatitis B Virus Core Protein and Empty Core-Like Particles and Induce Structural Changes
Source: PLoS Pathog. 2016 Aug 12;12(8):e1005802. doi: 10.1371/journal.ppat.1005802 (PMC4982637; doi:10.1371/journal.ppat.1005802)
Supplement: S5 Fig — The atomic model of HBV (PDB code 1QGT, color in red, green, cyan and yellow) was fitted into cryo-EM density of Cp183/Impβ complex prepared in 0.15M ammonium formate. The left half of each panel shows the 3D reconstruction computed from whole particles and the right half shows the 3D reconstruction computed from the dark particles. Enlarged views show the Impβ (PDB code 3LWW, color in blue) fitted into cryo-EM density map which rendered at the low contour level. Oval, triangle, and pentagon indicate locations of twofold, threefold and fivefold axes, respectively. The lower panel shows the difference map between the 3D reconstruction computed from whole particles (left) and the 3D reconstruction computed from the dark particles (right). This is essentially a featureless hollow sphere. The contour level was chosen to match the internal density within the 3D reconstruction computed with dark particles. To facilitate comparison, both maps were calculated at 15 Å resolution. As the Impβ density is heavily averaged and relatively weak, the fit in the inset is presented to allow the reader to gauge the amount of space available. (PDF) [file ppat.1005802.s005.pdf]

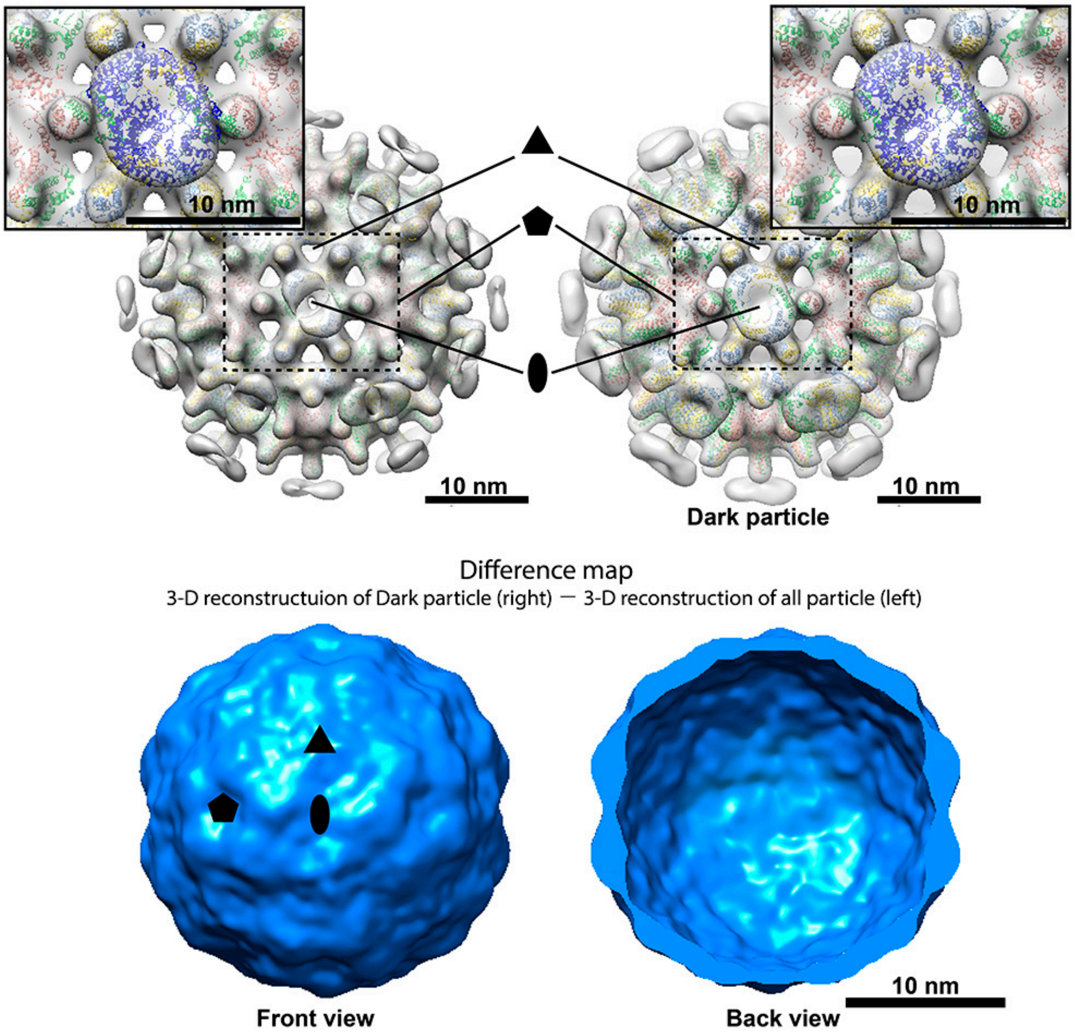

S5 Figure. Fitting atomic models into cryo-EM of the Cp183/Impβ complex (upper panel). The atomic model of HBV (PDB code 1QGT, color in red, green, cyan and yellow) was fitted into cryo-EM density of Cp183/Impβ complex prepared in 0.15M ammonium formate. The left half of each panel shows the 3D reconstruction computed from whole particles and the right half shows the 3D reconstruction computed from the dark particles. Enlarged views show the Impβ (PDB code 3LWW, color in blue) fitted into cryo-EM density map which rendered at the low contour level. Oval, triangle, and pentagon indicate locations of twofold, threefold and fivefold axes, respectively. The lower panel shows the difference map between the 3D reconstruction computed from whole particles (left) and the 3D reconstruction computed from the dark particles (right). This is essentially a featureless hollow sphere. The contour level was chosen to match the internal density within the 3D reconstruction computed from with dark particles. To facilitate comparison, both maps were calculated at 15 Å resolution. As the Impβ density is heavily averaged and relatively weak, the fit in the inset is presented to allow the reader to gauge the amount of space available.
