# Supplementary material for: Importin β Can Bind Hepatitis B Virus Core Protein and Empty Core-Like Particles and Induce Structural Changes
Source: PLoS Pathog. 2016 Aug 12;12(8):e1005802. doi: 10.1371/journal.ppat.1005802 (PMC4982637; doi:10.1371/journal.ppat.1005802)
Supplement: S6 Fig — The black (phosphorylated empty Cp183), green (phosphorylated Cp183 with Impβ) and red (Cp183 with Impβ) curves show the FSC for the 3D reconstructions in Fig 3. The blue curves (thick, all particles; thin, dark particles) show the FSC for the 3D reconstruction in Fig 7. The gray curves (thick, all particles; thin, dark particles) show the FSC for the 3D reconstruction in Fig 8. The 3D reconstruction of the dark particles in NaCl was calculated using gold-standard FSC. (PDF) [file ppat.1005802.s006.pdf]

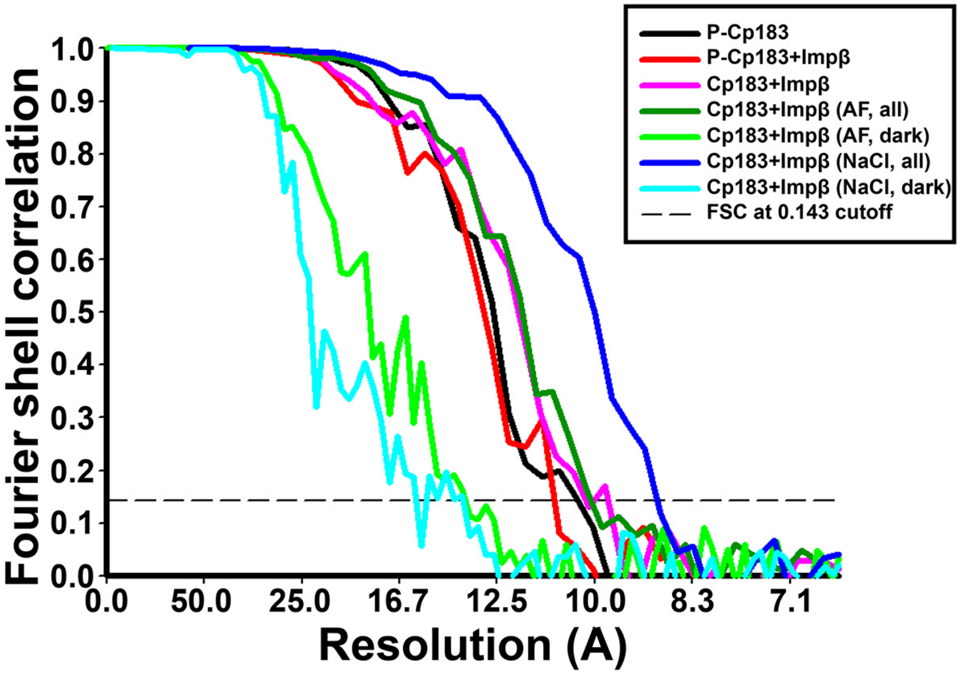

Figure S6. Resolution estimation. The black (phosphorylated empty Cp183), green (phosphorylated Cp183 with Impβ) and red (Cp183 with Impβ) curves show the FSC for the 3D reconstructions in Figure 3. The blue curves (thick, all particles; thin, dark particles) show the FSC for the 3D reconstruction in Figure 7. The gray curves (thick, all particles; thin, dark particles) show the FSC for the 3D reconstruction in Figure 8. The 3D reconstruction of the dark particles in NaCl was calculated using gold-standard FSC.
