# Supplementary material for: Importin β Can Bind Hepatitis B Virus Core Protein and Empty Core-Like Particles and Induce Structural Changes
Source: PLoS Pathog. 2016 Aug 12;12(8):e1005802. doi: 10.1371/journal.ppat.1005802 (PMC4982637; doi:10.1371/journal.ppat.1005802)
Supplement: S1 Table — As SEC is not an equilibrium technique, Impβ can dissociate during the course of the 40 minute-long experiment. In addition, some capsid-Impβ complex may precipitate before or during chromatography. These complications result in an underestimate of capsid-bound Impβ. (PDF) [file ppat.1005802.s007.pdf]

Table S1. Estimated Impβ : capsid ratio determined from size exclusion chromatography.

Panel	Sample	Content[1]	Salt[2]	impβ per capsid[3]
	5.3 μM Impβ 7.9 μM Cp			
2a	Cp183	-	.15M	40
2b	Cp183	-	.25M	4.5
2c	Cp183	RNA	.15M	-1
2d	P-Cp183	-	.15M	12
2e	P-Cp183	-	.25M	1.5
2f	P-Cp183	RNA	.15M	0.5
2g	Cp149	-	.15M	ND*
	18.8 μM Impβ, 11μM Cp			
6a	Cp183	-	.15M NH_4CO_2	26
6b	Cp183	-	.15M	30

1. Capsids are empty, in vitro assembled particles or RNA-containing particles from the E. coli expression system

2. Except for panel 6a, samples are buffered by 20mM TrisHCl pH 7.4. Panel 6a has 0.15M NH_4CO_2 pH 7.

3. Relative protein molar ratios were estimated from Coomassie stained gels using the program imageJ. Errors in background correction will be most evident in cases where the signal is weak.

* ND – Not Detectable. No detectable impb was observed to co-migrate with Cp149 in either Coomassie staining or silver staining.

As SEC is not an equilibrium technique, Impβ can dissociate during the course of the 40 minute-long experiment. In addition, some capsid-Impβ complex may precipitate before or during chromatography. These complications result in an underestimate of capsid-bound Impβ.
