# Supplementary material for: Importin β Can Bind Hepatitis B Virus Core Protein and Empty Core-Like Particles and Induce Structural Changes
Source: PLoS Pathog. 2016 Aug 12;12(8):e1005802. doi: 10.1371/journal.ppat.1005802 (PMC4982637; doi:10.1371/journal.ppat.1005802)
Supplement: S2 Table — Data are presented for seven reconstructions of the full-length core protein Cp183 with and without Impβ. (PDF) [file ppat.1005802.s008.pdf]

Table S2. Data collection information

Sample	Salt (M)	Nominal magnific-ation	Number of CCD images	Number of boxed particles	Number of particles in the final reconstruction	Defocus (µm)	Resolution (FSC[4])	EMDB accession numbers
P-Cp183	0.15 NaCl	40,000x	75	22,989	16,093	0.8 ~ 4.45	10.1Å	EMD-3266
P-Cp183/ Impβ[1]	0.15 NaCl	40,000x	81	7,200	5,760	1.07 ~ 4.36	10.9Å	EMD-3267
Cp183/ Impβ[1]	0.15 NaCl	80,000x	159	14,195	12,776	1.40 ~ 4.04	10.2Å	EMD-3268
Cp183/ Impβ[2]	0.15 AF[3]	80,000x	129	12,004	8,365	1.56 ~ 5.46	10.1Å	EMD-3269
Cp183/ Impβ[2] (dark particles)	0.15 AF[3]	80,000x	129	4,516	3,154	1.56 ~ 5.46	13.8Å	EMD-3270
Cp183/ Impβ[2]	0.15 NaCl	80,000x	109	7,225	6,443	0.91 ~ 4.57	8.9Å	EMD-3271
Cp183/ Impβ[2]	0.15 NaCl	80,000x	109	1,701	1,701	0.91 ~ 4.57	15.9Å	EMD-3272

1. 7.9 µM Cp183 dimer in capsid form and 5.3 µM Impβ

2. 11 µM Cp183 dimer in capsid form and 18.8 µM Impβ

3. AF, ammonium formate

4. Resolution for this sample was calculated using gold-standard Fourier shell correlation and is reported for a correlation at 0.143
